# Telehealth interventions for substance use disorders in low- and- middle income countries: A scoping review

**DOI:** 10.1371/journal.pdig.0000125

**Published:** 2022-11-02

**Authors:** Margaret Isioma Ojeahere, Sarah Kanana Kiburi, Paul Agbo, Rakesh Kumar, Florence Jaguga

**Affiliations:** 1 Department of Psychiatry, Jos University Teaching Hospital, Jos, Plateau State, Nigeria; 2 Department of Psychiatry, Mbagathi Hospital, Nairobi, Kenya; 3 Department of Psychiatry, Dalhatu Araf Specialist Hospital, Lafia, Nassarawa, Nigeria; 4 Department of Psychiatry & Deaddiction, G.G.S.M.C, Punjab, India; 5 Department of Mental Health, Moi Teaching & Referral Hospital, Eldoret, Kenya; University of Leeds, UNITED KINGDOM

## Abstract

The increasing prevalence and magnitude of harmful effects of substance use disorders (SUDs) in low- and middle-income countries (LMICs) make it imperative to embrace interventions which are acceptable, feasible, and effective in reducing this burden. Globally, the use of telehealth interventions is increasingly being explored as possible effective approaches in the management of SUDs. Using a scoping review of literature, this article summarizes and evaluates evidence for the acceptability, feasibility, and effectiveness of telehealth interventions for SUDs in LMICs. Searches were conducted in five bibliographic databases: PubMed, Psych INFO, Web of Science, Cumulative Index of Nursing and Allied Professionals and the Cochrane database of systematic review. Studies from LMICs which described a telehealth modality, identified at least one psychoactive substance use among participants, and methods that either compared outcomes using pre- and post-intervention data, treatment versus comparison groups, post-intervention data, behavioral or health outcome, and outcome of either acceptability, feasibility, and/or effectiveness were included. Data is presented in a narrative summary using charts, graphs, and tables. The search produced 39 articles across 14 countries which fulfilled our eligibility criteria over a period of 10 years (2010 to 2020). Research on this topic increased remarkably in the latter five years with the highest number of studies in 2019. The identified studies were heterogeneous in their methods and various telecommunication modalities were used to evaluate substance use disorder, with cigarette smoking as the most assessed. Most studies used quantitative methods. The highest number of included studies were from China and Brazil, and only two studies from Africa assessed telehealth interventions for SUDs. There has been an increasingly significant body of literature which evaluates telehealth interventions for SUDs in LMICs. Overall, telehealth interventions showed promising acceptability, feasibility, and effectiveness for SUDs. This article identifies gaps and strengths and suggests directions for future research.

## Author summary

Substance use disorders (SUDs) are an increasing public health problem which affects both children and adults across the world. Increasing numbers of individuals who live in low- and middle-income countries (LMICs) are affected by the worsening problems of SUDs compared to other regions. The wide treatment gap between people who suffer from the consequences of SUDs compared to those who have access to appropriate treatment ensures that out of reach populations and people in rural areas lack adequate healthcare for SUDs. Repeatedly, studies show that SUDs are chronic and recurring in nature. Therefore, affordable treatment innovations which integrate holistic strategies, promote self-monitoring and management approaches, that can be accessed by out of reach populations should be embraced. Although this may appear unrealistic and implausible in LMICs, telehealth intervention which is the use of communication technologies (text messages, phone calls, mobile applications, virtual reality to mention a few) to deliver healthcare across a distance has the potential to reduce this treatment gap. Our scoping review summarizes published studies which assessed the acceptability, feasibility, and effectiveness of telehealth interventions for SUDs in LMICs and highlights the gaps and directions for future research.

## Introduction

Substance use disorders (SUDs) are a growing public health concern of global significance with their impact cutting across all domains of life [1]. The 2021 world drug report of the United Nations Office on Drugs and Crime (UNODC) estimated that in 2019, 5.5% (275 million) of the global population (aged between 15 and 64 years) had used drugs and over 36 million people worldwide suffered from SUDs [2]. Using the World Bank country classification of low-and-middle income countries (LMICs) [3], increasing populations of people with the highest risk of substance use reside in LMICs and this is projected to increase by 40% in Africa by 2030 [4]. SUDs is one the world’s leading causes of years lived with disability in LMICs and its resulting consequences pose major challenges to health systems globally, particularly to those in LMICs [5].

There is a huge treatment gap for SUDs globally with one in six people who receive treatment for SUDs and one in 11 and 18 people who receive treatment in Latin America and Africa respectively [6]. Generally, the treatment gap for SUDs ranges from 75% to 95% in LMICs with higher values reported in rural areas [5,7,8]. Whilst SUDs prevalence have increased across the world, existing evidence shows that over 80 to 85% of people with mental disorders in LMICs do not have access to appropriate mental healthcare especially the out of reach populations [5]. Arguably, there is an overlap between people who require treatment for SUDs and other mental disorders [9]. Nevertheless, the worsening problems of SUDs continue to compound the existing unmet mental health needs [5,9].

A considerable number of studies show that SUDs can be chronic, relapsing disorders with cycles of relapses and remissions [1]. The chronicity of SUDs suggests that an established care model which provides an integrated care system consisting of self-management and services is required to prevent relapse in individuals diagnosed with SUDs [10,11]. Several approaches that address recovery management, improve continuity of care, monitor periods of abstinence, and early intervention, encourage self-management, mutual aid, other recovery supports, and system-level interventions have been recommended in the management of SUDs [12]. The principal caregivers of affected individuals are they themselves. Therefore, it becomes pertinent to explore interventions with potentials for self-monitoring and self-management with favorable outcomes and reduced economic burden [13]. Present management approaches for SUDs usually involve a combination of behavioral therapy, brain stimulation techniques, pharmacological therapies, and the use of telecommunication technologies [1]. One strategy that has been recommended with favorable outcome in addressing the chronicity of SUDs and improving access to care especially for out of reach populations is telehealth [14]. Conventionally, telehealth interventions involve the use of communication technologies to deliver healthcare across a distance [15].

Emerging evidence shows that telehealth has the potential of reducing the existing treatment gap in LMICs in the diagnosis and management of people with mental disorders and SUDs. These interventions range from simple and easily accessible forms such as text messaging, and phone calls, to more advanced modalities such as virtual reality, videoconferencing and the use of innovative web-based platforms and mobile apps [16]. This form of healthcare intervention has been in existence for over half a century but it remains underutilized in several LMICs [17]. Several factors such as user barriers (awareness, level of education, availability of gadgets, affordability of resources, telehealth literacy), organizational, and program barriers are contributory to its limited use in LMICs [18]. On the other hand, telehealth interventions have been better endorsed and used in high income countries (HICs) with most studies assessing use of these interventions for substances such as alcohol use, cannabis, tobacco with limited use for opioid use disorder and methamphetamines [19–22]. These interventions have been shown to be effective in improving substance use outcomes and other outcomes such as quality of life [23,24]. Additionally, participants reported high satisfaction with use [25,26]. In LMICs there is limited use of digital interventions in SUDs treatment, for example, a systematic review on the use of digital interventions for mental health treatment and prevention identified only six studies who utilized telehealth interventions for SUDs out of the 49 articles [27]. An update of this review in 2021 identified seven articles on SUDs from LMICs [28]. Among the challenges cited with the use of telehealth interventions for SUDs treatment in LMICs include high rate of technology evolvement [29], limited access to internet and cost of airtime, lack or poor literacy skills needed to access these interventions, lack of non-verbal cues, high phone turnover, privacy, and litigation concerns [30–32].

Growing evidence demonstrates that remote management by means of telecommunications technology, offers a promising approach in improving accessibility and affordability of care of individuals with SUDs and has the prospect of facilitating self-monitoring and management of individuals with SUDs [33,34]. Several authors have examined feasibility, acceptability, and effectiveness of telehealth interventions for SUDs worldwide and have suggested different definitions for these. Overall, feasibility as a construct in public health practice takes into cognizance several aspects of intervention delivery. These include demand (is the intervention taken up?), implementation (can it be delivered as planned?), practicality (can it be delivered despite constraints, such as resources and time?). Feasibility incorporates acceptability, i.e how the recipients of (or those delivering) the intervention perceive and react to it [35].

There is a dearth of literature reviews on telehealth interventions for SUDs in LMICs. To address the gap of research on this topic, we conducted a scoping review of available literature on this subject to provide a preliminary overview to identify existing gaps from the available evidence, and to describe trends on this topic while addressing it from a broader perspective, unlike systematic review which develops critically appraised and synthesized results. Therefore, the objectives of this scoping review were to summarize literature evaluating the acceptability, feasibility, and effectiveness of telehealth interventions for SUDs in LMICs, to identify evidence gaps and proffer recommendations for future research.

## Materials and methods

Following the conception of the topic, an exploratory search was carried out to determine the extent of literature on telehealth interventions for SUDs in LMICs, guided by formulation of the review question and identification of key concepts, search terms, phrase strategy and testing of the search strategy.

### Protocol and registration

There was no formal registration of this scoping review with the international systematic review database (PROSPERO). As of the time of writing this manuscript, it was not a requirement for scoping reviews to be registered with PROSPERO. The Quality Assessment Tool for Studies with Diverse Designs (QATSDD) [36], was employed in assessing the quality of studies reviewed. Although, as of the time of writing, scoping reviews do not typically require quality assessment unlike systematic review which generally requires quality assessment of included studies [37].

The method used in this review adopted the framework developed by Arksey and O’Malley [38], and modified by Levac and colleagues [39], and the Joanna Briggs Institute [40]. Consistent with this method, the scoping review was conducted in 5 main stages: Developing the research question; identifying relevant studies; literature selection; charting the data; and collating, summarizing, and reporting the results.

#### Stage 1: Developing the research question

We developed a broad research question for our literature search, asking what the academic literature says about the acceptability, effectiveness, and feasibility of telehealth interventions for SUDs in LMICs.

#### Stage 2: Identifying relevant studies

Search strategy: Five different electronic databases: PubMed, PsychINFO, Web of Science, Cumulative Index of Nursing and Allied Professionals (CINAHL) and Cochrane Library were used to search for articles published in English or translated to English to identify relevant studies. Different search engines were engaged and the initial database searches were conducted from September 30 to October 1, 2020 (S1 Data). Our searches spanned articles published from 2010 to time of search in 2020. A 10-year timeline was agreed upon by the authors because the use of digital intervention in LMICs gained prominence in the last decade.

The keywords used for the search in this review were “telehealth OR telepsychiatry OR telemedicine OR teleconsultation OR mobile health OR mhealth OR mobile phone OR web OR video conferencing OR SMS OR short message OR internet OR Substance use OR Substance use disorder OR substance abuse OR substance dependence OR addiction OR addict OR alcohol use disorder OR alcohol abuse OR alcohol dependence OR alcohol addiction OR tobacco OR cigarette OR smoking OR nicotine OR cannabis OR marijuana OR bhang OR Khat OR shisha OR heroin OR opioid OR injecting drug use OR people with injecting drug use OR PWID OR cocaine OR amphetamine OR methamphetamine OR Feasibility AND Effectiveness”. (S1 Table)

Manual extraction of relevant literature from the reference list of articles included in the review was done. This entailed consideration of relevant terms, dates, text words contained in the title, abstracts of retrieved papers and index terms. The PICOS (participants, intervention, context, outcomes, and study design) framework [41], was used to establish eligibility criteria.

*Inclusion criteria*. We included articles that examined at least one of the following outcomes such as acceptability, feasibility, effectiveness of telehealth interventions for substance use if: (a) the population examined or part of the population was from an LMICs as defined according to World Bank country classification (b) the article was an original research (c) there was evidence of substance use exposure, (d) articles were published in English or had an English translation available, (e) the studies was conducted among all age groups (f) studies used all designs quantitative, qualitative and/or mixed. (g) there was evidence of a substance use/SUDs related intervention outcome such as acceptability/ feasibility and/or effectiveness.

In this study, feasibility was defined in broad terms and included variables such as: ease of recruitment of participants, number of participants recruited in relation to targeted sample size, cost effectiveness, ease of delivery of the telehealth intervention, retention in the program follow up, and acceptability (perceived usefulness of the intervention, ratio of participants who dropped out/ requested to be removed from program, likability of the intervention and willingness to recommend intervention to others) [42–46]. For this review, articles that assessed ‘Effectiveness’ were those that described change in substance use following the intervention. Change in substance use was assessed through self-report, use of standardized tools or criteria and biochemical tests [44–46].

*Exclusion criteria*. Studies were excluded if: (a) they were conducted across LMIC and HICs and did not report LMIC specific results (b) they were review articles, dissertations, conference presentations or abstracts, case studies, commentaries, editorials, or grey literature (c) the full text articles were not available.

#### Stage 3: Literature selection

Following the search, all articles identified were exported to Mendeley reference manager where the initial removal of duplicates was done. Next, they were exported onto Rayyan (a software for screening and selecting studies for systematic and scoping reviews and detecting duplicates) [47], whereby, after further removal of duplicates, the abstracts and titles of retrieved articles were independently screened by two authors (M.O and F.J) based on the predetermined eligibility criteria for inclusion in the full text screening. A second screening of full text articles was also done independently by two other authors (S.K and R.K) and resulted in an 85% agreement. Disagreements during each stage of the screening were resolved through discussion and consensus. In instances where consensus could not be reached, a third author was invited to review. Screening of selected studies was performed between October 2, 2020 to March 30, 2021.

#### Stage 4: Charting the data

A comprehensive data extraction form was prepared in Microsoft Excel by the authors. The form was first piloted by F.J and S.K on ten articles to ensure consistency and necessary adjustments were made to the content thereafter. Data was extracted by all authors and the final form was entered by M.O and double checked by M.O and S.K for completeness and accuracy. The draft of the manuscript was written by M.O and discrepancies were resolved by discussion with S.K and F.J until consensus was achieved. The following data were extracted: author, year; country; modality of telehealth intervention; targeted substance; study design; sample size; study setting/population; measures of acceptability, feasibility, effectiveness, and other outcomes. After familiarization with the data, two authors (M.O and F.J) inductively identified seven specific themes from the data which were reviewed and affirmed by the other authors.

#### Stage 5: collating, summarizing, and reporting the results

A narrative account of the included articles was prepared to present patterns in telehealth interventions as acceptable/ feasible and/ or effective tools or not applicable in the reduction of SUDs in LMICs. The results have been summarized descriptively and defined by the authors to alleviate the narrative account and grouped into the following emergent themes: *publication trends/ timing of publication*, *country of research*, *population and setting*, *research design*, *psychoactive substance of interest*, *telehealth modality utilized*, *measured outcome* (S2 Table).

## Results

### Search results

**Fig 1:**
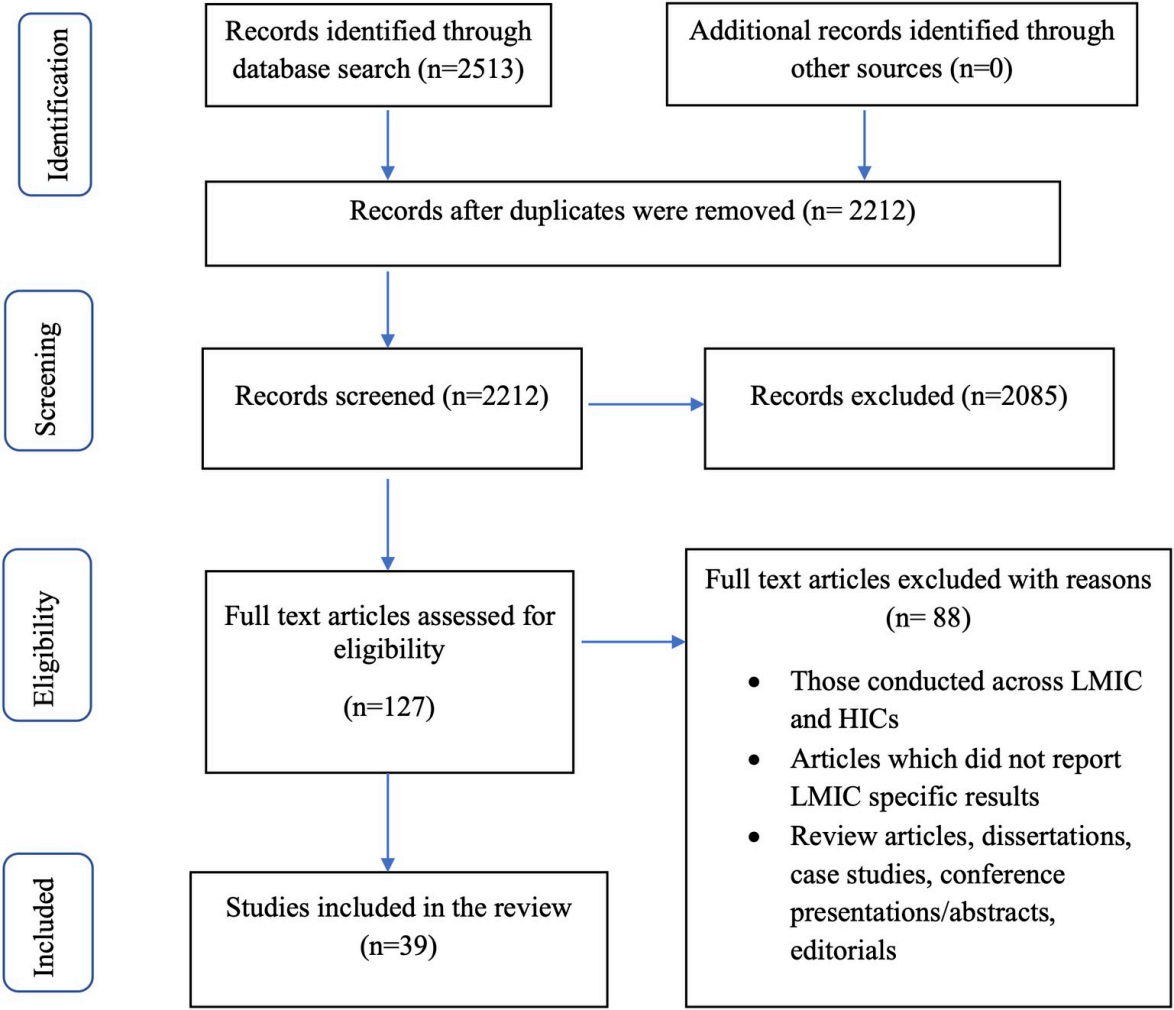
PRISMA Flowchart describing the selection of studies mapping existing literature on acceptability, feasibility, and effectiveness of telehealth interventions for SUDs in LMICs.

Our search in five electronic databases identified a total of 2513 articles through PubMed (1978), Psych INFO via EBSCOhost (128), Web of Science (108), Cumulative Index of Nursing and Allied Professionals via EBSCOhost (CINAHL) (60), and Cochrane Library (239). A total of 301 duplicated articles were excluded and 2212 articles underwent title and abstract screening. A total of 2085 publications which did not meet the inclusion and exclusion criteria were excluded and 127 full-text articles were retained. Following full text review, 39 articles were included in the data extraction (Fig 1).

This method of research was conducted on the basis of a predetermined protocol in accordance with the Preferred Reporting Items for Systematic reviews and Meta-Analyses (PRISMA) standards for scoping reviews [48] (S1 PRISMA Checklist).

### General characteristics of included studies

Original peer reviewed articles that examined the topic have progressively increased in the last 10 years with 37 articles (94.9%) published in the last 5 years of this review (2016–2020). The year 2019 had the highest frequency of identified publications, n = 12 (30.8%).

### Publication trends/ timing of publication

**Fig 2:**
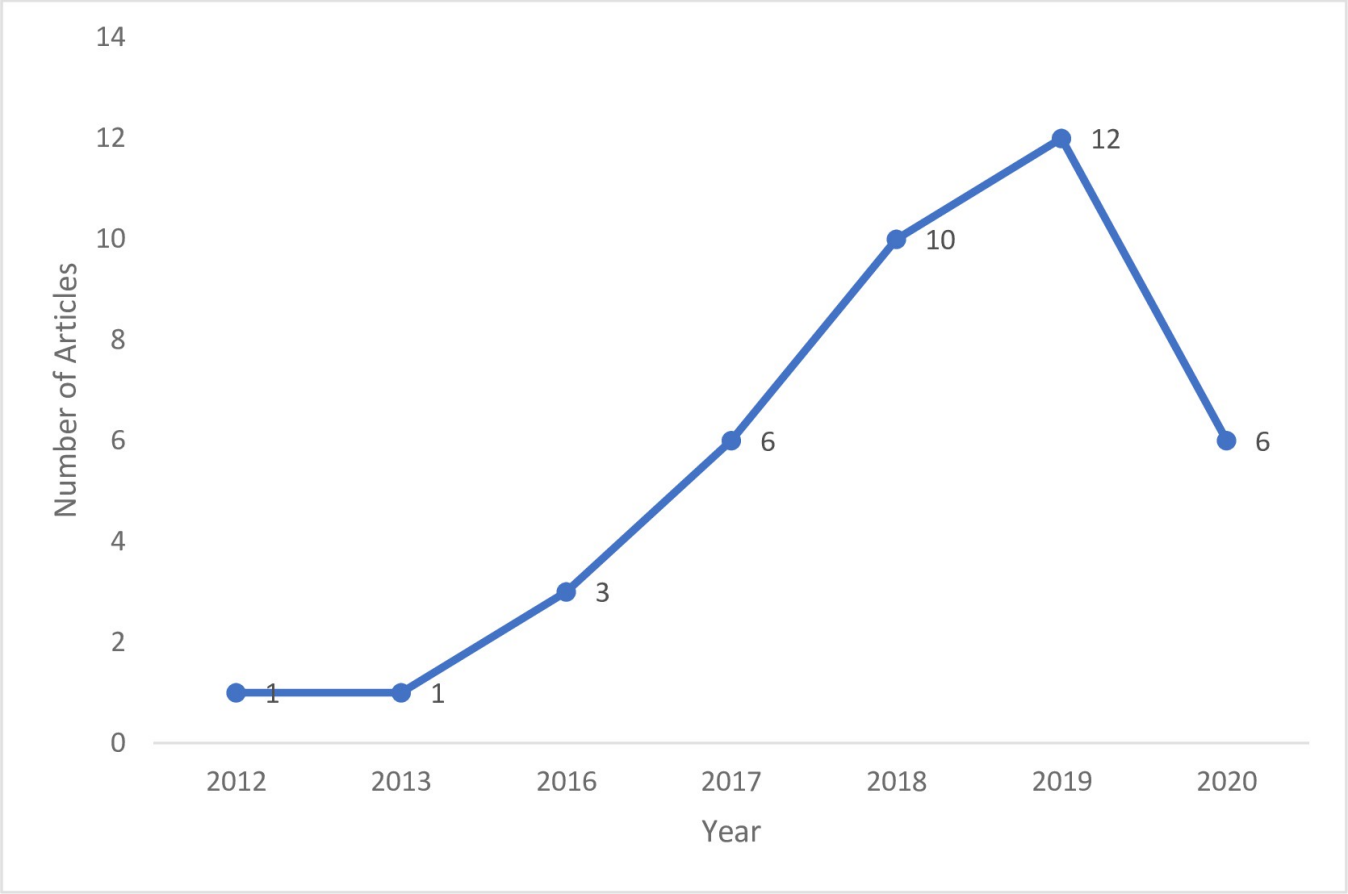
Line graph showing articles published per year (publication trends as of time of data collection).

The earliest identified study which met the eligibility criteria was conducted in 2012 [49]. One study each was found in 2012 and 2013 [46,49]. Our review identified three publications in 2016 [50–52], and six articles in 2017 [53–58]. Thereafter, the number of studies increased with a total of 28 publications between 2018 and 2020. Ten publications were found in 2018 [13,59–67]. The highest proportion of included literature which met the eligibility criteria for this review was published in 2019 with 12 articles [44,68–78] and six published articles were found in 2020 [16,45,79–82] (Fig 2).

### Country of research

**Fig 3:**
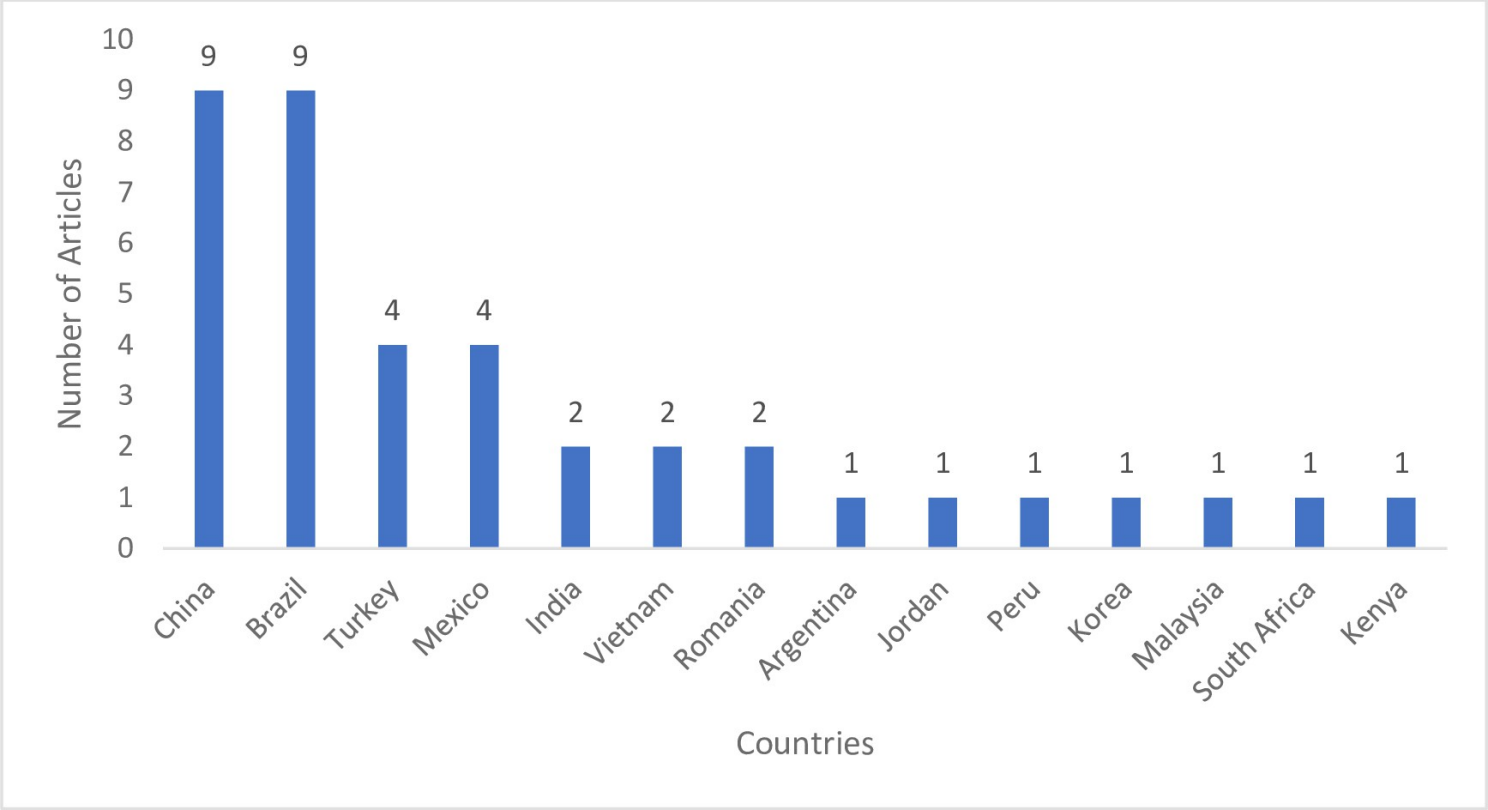
Bar chart showing the distribution of articles per countries.

A total of 14 countries were represented in this review. The country with the highest number of included studies was China [44,45,53,54,57,59,60,62,67] and Brazil [13,56,63,64,66,70,72,73,79] with a total of nine studies each. Turkey [46,49,50,71] and Mexico [65,68,69,82] had four publications each. India [52,76], Vietnam [61,81], and Romania [55,78] had two studies each. Other countries such as Argentina [16], Jordan [51], Peru [58], Malaysia [74], Korea [75], South Africa [77], and Kenya [80] had one article each (Fig 3).

### Population and settings

The age range of participants in the included studies was 12–87 years [63,68]. Across studies identified, four focused on children and adolescents [55,63,74,78], and two had 15 and 17 years as their lower age limit [61,65]. Two studies were on only male patients [53,62], one on parents of adolescents [66], and one on smoking fathers, non-smoking mothers, and exposure of their newborns to secondhand smoking (SHS) [54]. Our review identified a heterogeneous distribution of participants across studies. Participants cut across individuals in the community who were dependent on psychoactive substances and indicated a willingness to quit, oil workers, night club patrons, families, and children and adolescents. Consequently, settings spanned across workplace [75], general population [16,45,46,49,52,56–59,66,81,82], health treatment facilities [44,50,51,53,54,60,65,67–71,76,77,79,80], night clubs [13,64], and schools [55,61,63,72–74,78].

### Study design and sample size

The selected articles in this review used quantitative, qualitative, and mixed methods. Most studies primarily used quantitative methods (n = 30). Sample sizes for individuals ranged from 40 to 23054 [62,72]. One study was done prior to implementation of the intervention to assess the willingness to use a mobile phone application for smoking cessation [61]. Four studies used qualitative methods [58,66,77,81]. Two studies reported an ecological momentary assessment (EMA) approach to examine psychoactive substance and exposure to social and environmental cues [52,67]. Secondary data analysis was used in three identified studies [67,78,82].

### Psychoactive substance of interest

Studies which assessed cigarette smoking across various populations dominated our review with 25 articles [16,45,46,49–52,54,55,57–59,61,63,65,68–71,74,75,78,79,81,82], followed by alcohol use in eight articles [13,44,56,64,72,73,76,80]. One study assessed only methamphetamine [62], one assessed any psychoactive substance use [66], and one study assessed both cigarette smoking and alcohol [77]. Three studies assessed multiple drug use such as heroin or amphetamine, opioid use, methamphetamine [60,65,67].

### Telehealth modality utilized

Studies included in this review used either one or a combination of telehealth interventions such as SMS, telephone calls, web-based cessation programs, mobile applications, web-based surveys, emails, virtual reality, hybrid phone counselling, and /or one-on-one consultations. Eleven studies used short message system (SMS) [46,49–51,54,57,59,68,75,77,81], and twelve studies incorporated web based mobile interventions programs [44,55,56,64,65,68,69,72–74,78,79]. Nine of the articles used mobile apps such as “smoker face”,” WeChat”, “WhatsApp”, and S-health [16,44,59,60,62,63,67,80,82]. A considerable number of studies utilized telephone communication [13,53,64,66,75,76], and some studies applied hybrid forms in delivering the interventions [13,64,68,70,75]. Psychotherapy and pharmacological interventions were used in addition to telehealth intervention in some studies [44,79,80]. The telehealth modalities were delivered via digital platforms and face to face consultations. Feedback from the telehealth modalities utilized were majorly asynchronous and engaged the services of medical students, trained counselors, healthcare providers, and researchers were employed for the delivery of the respective interventions. Of the 31 quantitative studies, twenty-nine publications clearly stated how the intervention was administered. Two studies were majorly automated messages [49,64]. Two others did not report who delivered the intervention [13,50] and one did not implement the intervention on participants [61]. Five studies clearly reported that trained counsellors in substance cessation treatments were engaged to deliver the intervention [53,54,65,68,70]. A heterogenous spread of physicians and allied professionals such as clinicians with masters/ degree in nursing/ doctoral degree in clinical psychology/ medical degree and trained in mental disorders/ occupational nurse with over 10 years’ experience provided face to face consultations in three studies [44,75,80], and one was delivered by medical students [63].

Although the heterogeneity of the frequency and duration of sessions made it difficult to combine, varying durations of sessions which ranged between 5mins to 60mins across studies were recorded in our review [65,72]. The number of sessions of the telehealth interventions conducted ranged between 1 to 42 sessions usually at baseline and at predefined times in the program [16,68]. Eight authors conducted one session each in the course of their study [63,68–71,74,79,80]. Three sessions were recorded by Bedendo and colleagues [68], and daily interactions for 4 weeks was reported in a study by Liang and colleagues [60]. Other studies reported a minimum of 4, 5 and 6 sessions [46,53,55], weekly sessions of 6 and 12, and 20 sessions were conducted by other authors [44,45,62].

### Measured outcomes

Adherence to treatment was assessed in all the 31 quantitative studies identified and rates of adherence to protocol ranged from 45% to 100% [53,71]. Higher adherence rates of 74.6% to 100% were seen for cessation of smoking among participants [59,71]. Increased adherence rates were seen with the usage of WhatsApp, text messages, web based and mobile applications. One study reported poor agreement between mobile application, multiple substances assessed and laboratory findings [67].

Acceptability as described earlier was recorded in 17 of the selected studies. A range of 51% to 99.3% favorable reactions to the program were observed among those who provided data on acceptability [46,63]. Acceptability was not clearly stated or assessed in 22 identified studies but findings were generally suggestive of a successful program and recommended for future use [13,44,50–52,54,56–59,62,64,65,67,71–73,75,76,78,80,82]. Most patients rated the intervention sessions as helpful or very helpful and about half of the patients reported reduction in cigarette smoking and alcohol use [66,77,80].

Effectiveness was assessed in 37 of the selected publications and several identified studies showed promising efficacy after preliminary results [16,61,62,68,76]. Twelve studies reported their outcome as both feasible and effective [44,45,50,52,56–59,63,64,72,75].

Our review identified four qualitative studies [58,66,77,81]. Generally, there was an overall high interest in those with intention to quit among participants. Findings from the focus group discussions and in-depth interviews demonstrated that the majority of participants wanted to quit smoking but did not have a plan, some wanted more digital reminders but all the participants found the intervention to be helpful [58,81]. Some authors reported no significant differences before and after the intervention [53,65,80]. However, only a few authors reported poor agreement between the type of mobile application used, substance assessed and laboratory findings as well as poor outcomes seen with high drop-out rates, low post intervention rates, no significant reduction in the substance use [13,45,46,53,54,67,73,74,78].

## Discussion

This scoping review aimed to provide an overview of literature examining the acceptability, feasibility, and effectiveness of telehealth interventions in the management of SUDs in LMICs and to highlight areas of gaps in research on this subject.

Overall, our review identified seven main themes. Our findings show that considerable work has been done on the treatment of SUDs using telehealth interventions globally but with only a few studies from LMICs and fewer yet from Africa. The earliest identified study was in 2012 [49], and research on this topic has progressively increased over the last 10 years with the highest number of studies seen in 2019 as at the time of data extraction. This suggests that more clinicians and researchers in LMICs are realizing the role of telehealth interventions in addressing SUDs and are embracing this innovative method of healthcare delivery and this can also be related to increase in availability and use of information technology in recent years [65,69,79,83,84]. However, in the face of the importance of this subject and the existing evidence from HIC, there is a paucity of research in LMICs [46,85]. Amongst the 14 countries represented in this review, China and Brazil have the highest number of studies. A plausible explanation for this may be because China has the highest mobile phone and internet users in the world with an estimated number of 1.02 billion internet users as of January 2022 [86]. Similarly, Brazil has experienced an increasing internet user in the last decade with over 140 million and 167.7 million number of users on the web (with the latter equivalent to 77.87 percent of the country’s population) as of 2018 and 2022 respectively. The smartphone, which was one of the most commonly used devices as of 2021, among approximately 75.6% of the Brazilian population (within the 18–55-year-old age group) [87], may also be contributory.

Plausible factors for few or no entries of some LMICs include: factors such as limited/ no internet access to telecommunication technology especially in rural areas, internet illiteracy, worsening poverty levels and paucity of research from sub-Saharan Africa [5,7,43]. Arguably, the consequences of limited/ no resources or poor prioritization for research in SUDs cessation programs in some LMICs may explain the paucity of data. For example, a country like Nigeria with the highest number of internet users in Africa estimated at 109 million as of January 2022, was not represented in our review [86]. These suggest that telehealth interventions may be implemented for SUDs in LMICs but may go unreported or unpublished.

### Age

Majority of included literature were among adults with only four studies with their foci on children and adolescents [55,63,74,78]. Existential evidence is indicative of young adults as the fastest growing users of psychoactive substances across the world and an increasingly faster rate of substance use among individuals aged 40 years and above compared to younger populations [88]. Findings from our review suggest that adolescents can easily be motivated to abstain from psychoactive substances using telehealth applications [64,74]. Most people who start smoking in early adolescence are usually curious, fascinated by the practice and influenced by peer pressure [63,74], an intervention acceptable to peers in this population would have increased likelihood of favorable outcome. Considering that children and adolescents are in a phase in life when appearance is of great importance, therefore in mitigating SUDs, innovations which utilizes appearance as a telehealth tool as a school-based program will help to reduce the number of adolescents who eventually develop SUDs [74].

### Population setting

Our review shows that engaging the use of telehealth interventions early in primary care is feasible and effective in addressing SUDs in healthcare facilities evidenced by several studies in this review. Its flexibility enables its use in the workplace, nightclubs, schools and among families with promising results. In the assessment of SHS among smoking fathers, non-smoking mothers and their newborn in a parental program, Yu and colleagues reported increased abstinence rates and success in the reduction to exposure to smoke [54]. SUD is a public health problem which affects the individual, the family and society [1], consequently, SUD cessation programs which target the family and establish structures that protect children and adolescents in such environments should be encouraged. The majority of the reviewed studies assessed participants in urban settings, this underscores the need to promote telehealth interventions for hard-to-reach populations. The use of technology which provides opportunities for cost effective, improved healthcare and reaching underserved populations and prioritization for such programs should be encouraged in LMICs.

### Study design

Studies differed in interventions and approaches across different domains (sample size, settings, participants, and modalities). The bulk of studies identified in this review used quantitative study design and the majority were randomized controlled trials (RCTs). Identified articles assessed interventions at individual and group levels and majority of studies examined interventions and comparison groups which added weight to such studies. Our review suggests that preliminary results were acceptable, feasible and effective. Mechanisms which contributed to significant findings had a diverse spread and studies which utilized eclectic approaches may require subsequent replication across settings to ascertain positive outcomes of telehealth interventions in the management of SUDs [54,69]. Findings from the qualitative studies show that the majority of participants enjoyed the sessions but they reported that they did not like the counseling sessions being recorded and similar reports were noted by the facilitators who felt recording the session hindered ease of participation. Overall, small sample size, insufficient power to definitively test the intervention, possible bias from self-reporting, absence of a comparison group, lack of regular follow-up, problems with technology, and high attrition rates, were some of the methodological limitations identified in our review [46,64,65,68,69,72,73].

### Psychoactive substance

Selected studies in this review assessed telehealth interventions for tobacco, alcohol, cannabis, opioids, cocaine, methamphetamine, MDMA, inhalants, hallucinogen, sedatives, and other psychoactive substances. A great proportion of the included studies examined telehealth interventions with regards to cigarette smoking, possibly because tobacco use is a major cause of disease burden and one of the top five preventable causes of death [89]. Studies which assessed multiple psychoactive substances recommended careful interpretation of results and caution in the adoption of results [65,67]. Overall, our review highlighted substantial reduction in psychoactive substances with telehealth interventions.

### Telehealth modality/measured outcomes

Mobile phone text messages are an affordable and effective way of overcoming resource barriers in LMICs, they have the potential of reaching a wide range of people, those in hard-to-reach areas and people who prefer non-face to face consultations and reducing stigma [44,70,79]. This may explain why most studies in this review utilized SMS as the telehealth intervention of interest. Several studies found text messages to be affordable and feasible mobile health intervention modalities with promising effectiveness in several of the studies reviewed [51,56,58,82]. Mobile applications were demonstrated to be effective in reducing stigma and surmounting barriers to accessing treatment in LMICs [45,79,80]. An innovative approach with promising effectiveness in addressing the barriers encountered in resource constrained settings is web-based information modality [28,56,69,90,91]. This form of telehealth intervention can be harnessed to provide information and health care services which targets reduction in SUDs and treatment strategies across a wide population of people [65,68,69,70], although, some authors reported high rates of attrition using this method [64,72,73]. Studies conducted among students in high schools and colleges which used a particular facial aging apps (“smoker face”) reported this intervention as acceptable [63,78]. Participants stated their preference for this app and described it as interesting [63,78]. The engaging nature and practicality of the app may be plausible reasons for its recommendation by participants. In evaluating the outcome of included studies, a great number of these studies demonstrated acceptability and feasibility as favorable outcomes and a considerable number showed promising effectiveness of telehealth interventions in the management of SUDs. However, not all authors found mobile apps and other telehealth modalities to be acceptable in the management of SUDs [46,65,67]. Cultural practices, existing preconceptions, confidentiality, and privacy issues were possible explanations for the poor acceptability of mobile health interventions [18,54,61,65]. Furthermore, lack of incentives, poor internet literacy, and limited or lack of internet connections especially in the rural areas were some of the factors identified as barriers to feasibility and effectiveness by participants [18,61,64,72,81].

### Strengths and limitations

The strengths for this scoping review include being one of the few that have examined outcomes of telehealth intervention for SUD treatment in LMICs, use of a transparent and reproducible process which states the search strategy, data sources, and data extraction. Our findings show that a great number of articles have been published on SUDs but only a small proportion of publications screened in comparison to the total number of selected articles assessed telehealth interventions as possible feasible, acceptable, and effective tools for SUDs in LMICs.

In addition to the above, our scoping review identified several limitations which include the exclusion of grey literature and evaluation of bibliographic databases and journals published in the English language, this may have resulted in relevant articles published in other languages to be overlooked. Additionally, not all authors we contacted for the full text of their studies responded. The study populations of the included studies examined circumscribed populations, and sample sizes which may make it difficult to generalize results. Our review aimed to provide an overview of literature published on telehealth interventions and SUDs and to identify gaps in research. Consequently, it is likely that the heterogeneity of methods used across studies may have affected the results reported.

## Conclusions

The present scoping review adds to the body of knowledge, provides a summary of findings on application of telehealth interventions in SUDs treatment in LMICs and underscores gaps in research and areas of emphasis. These findings can guide subsequent research and interventions geared at reduction of SUDs to improve outcomes. The increasingly innovative mobile health technology can provide opportunities for underserved populations. Existing evidence suggests its potentials in the reduction of SUDs if appropriately implemented. The evidence base is growing, although there is a gap of knowledge in literature examining the effect of telehealth interventions in the reduction of SUDs in LMICs. Therefore, it is difficult to draw a firm conclusion on its effectiveness. Future studies with larger scale randomized studies are required to evaluate the effectiveness of telehealth interventions for individuals with SUDs in LMICs.

Our recommendations include provision of resources and enabling conditions for telecommunication technology to thrive as an interventional health care tool in resource poor settings, prioritizing research in SUDs cessation programs and promoting publications of such activities. Future research is recommended on populations with larger sample size, longer follow up and replication of these methods across different populations to determine if telehealth interventions are as effective as some preliminary studies suggest. We also recommend that other unexplored outcomes of telehealth intervention such as issues around privacy, confidentiality, and cultural applicability of these methods of service delivery in LMICs should be addressed in subsequent research. Considering that adolescents are a significant proportion of those who use psychoactive substances [68], we recommend more research in this population with regards to SUDs. The incorporation of smoking/ substance use cessation programs into school programs should be supported. In addition, SUDs cessation programs using telehealth targeting children and parents should be considered as focus for future observational studies and in the establishment of favorable policies.

## Supporting information

S1 PRISMA Checklist PRISMA-ScR Checklist. (TIF)

S1 Data Database searches. (ZIP)

S1 Table Search String. (DOCX)

S2 Table Tables of Selected Articles. (DOCX)

## References

[1] Ibrahim C, Rubin-Kahana DS, Pushparaj A, Musiol M, Blumberger DM, Daskalakis ZJ, et al. The Insula: A Brain Stimulation Target for the Treatment of Addiction. *Front Pharmacol*. 2019; 10:720. doi: 10.3389/fphar.2019.00720 31312138 PMC6614510

[2] UNODC World Drug Report 2021: pandemic ramps up drug risks as youth underestimate cannabis dangers. June 24, 2021. Available from: https://www.unodc.org/unodc/press/releases/2021/June/unodc-world-drug-report-2021_-pandemic-effects-ramp-up-drug-risks—as-youth-underestimate-cannabis-dangers.html

[3] World Bank. World Bank country and lending groups, country classification. Washington (DC): World Bank. 2019. Available from: https://datahelpdesk.worldbank.org/knowledgebase/articles/906519-world-bank-country-and-lending-groups

[4] World drug report 2021. https://wdr.unodc.org/ United Nations Office on Drugs and Crime (UNODC). 2021. Available from: https://wdr.unodc.org/

[5] Heijdra Suasnabar J.M., Hipple Walters B. Community-based psychosocial substance use disorder interventions in low-and-middle-income countries: a narrative literature review. *Int J Ment Health Syst*.2020; 14:74. doi: 10.1186/s13033-020-00405-3 33062049 PMC7542947

[6] UNODC World Drug Report 2019: 35 million people worldwide suffer from drug use June 2019. Available from: https://www.unodc.org/unodc/en/frontpage/2019/June/world-drug-report-2019_-35-million-people-worldwide-suffer-from-drug-use-disorders-while-only-1-in-7-people-receive-treatment.html

[7] Rathod SD, De Silva MJ, Ssebunnya J, Breuer E, Murhar V, Luitel NP, et al. Treatment Contact Coverage for Probable Depressive and Probable Alcohol Use Disorders in Four Low- and Middle-Income Country Districts: The PRIME Cross-Sectional Community Surveys. *PloS one*. 2016; 11:9. e0162038. doi: 10.1371/journal.pone.0162038 27632166 PMC5025033

[8] Zewdu S, Hanlon C, Fekadu A, Medhin G, Teferra S. Treatment gap, help-seeking, stigma and magnitude of alcohol use disorder in rural Ethiopia. *Subst Abus Treat Prev Policy*. 2019;14(1):1–11. doi: 10.1186/s13011-019-0192-7 30658659 PMC6339347

[9] Otasowie J. Co-occurring mental disorder and substance use disorder in young people: aetiology, assessment and treatment. *BJPsych Advances*. 2021;27(4):272–281. doi: 10.1192/bja.2020.64

[10] Johnson K, Richards S, Chih MY, Moon TJ, Curtis H, Gustafson DH. A Pilot Test of a Mobile App for Drug Court Participants. *Subst Abuse*. 2016;10:1–7. Published 2016 Feb 14. doi: 10.4137/SART.S33390 26917964 PMC4755700

[11] Lorig KR, Sobel DS, Stewart AL, et al. Evidence suggesting that a chronic disease self-management program can improve health status while reducing hospitalization: a randomized trial. *Med Care*. 1999;37(1):5–14. doi: 10.1097/00005650-199901000-00003 10413387

[12] Dennis M, Scott CK. Managing addiction as a chronic condition. *Addict Sci Clin Pract*. 2007;4(1):45–55. doi: 10.1151/ascp074145 18292710 PMC2797101

[13] Baldin YC, Sanudo A, Sanchez ZM. Effectiveness of a web-based intervention in reducing binge drinking among nightclub patrons. *Rev Saude Publica*. 2018;52:2. doi: 10.11606/s1518-8787.2018052000281 29364357 PMC5777341

[14] Tuckson RV, Edmunds M, Hodgkins ML. *Telehealth*. *N Engl J Med*. 2017;377(16):1585–1592. doi: 10.1056/NEJMsr1503323 29045204

[15] Weinstein RS, Lopez AM, Joseph BA, Erps KA, Holcomb M, Barker GP, et al. Telemedicine, telehealth, and mobile health applications that work: opportunities and barriers. *Am J Med*. 2014;127(3):183–187. doi: 10.1016/j.amjmed.2013.09.032 24384059

[16] Goldenhersch E, Thrul J, Ungaretti J, Rosencovich N, Waitman C, Ceberio MR. Virtual Reality Smartphone-Based Intervention for Smoking Cessation: Pilot Randomized Controlled Trial on Initial Clinical Efficacy and Adherence. *J Med Internet Res*. 2020;22(7):e17571. doi: 10.2196/17571 32723722 PMC7424475

[17] Scott R, Mars M. Telehealth in the developing world: current status and future prospects. *Smart Homecare Technology and TeleHealth*. 2015;3:25–37. doi: 10.2147/SHTT.S75184

[18] Sagaro GG, Battineni G, Amenta F (2020) Barriers to sustainable telemedicine implementation in Ethiopia: A systematic review. *Telemedicine Reports*. 1:1, 8–15. 10.1089/tmr.2020.0002 35722252 PMC8812291

[19] Hoch E, Preuss UW, Ferri M, Simon R. Digital Interventions for Problematic Cannabis Users in Non-Clinical Settings: Findings from a Systematic Review and Meta-Analysis [published correction appears in Eur Addict Res. 2016;22(5):286]. *Eur Addict Res*. 2016;22(5):233–242. doi: 10.1159/000445716 27160333

[20] Ramsey AT, Satterfield JM, Gerke DR, Proctor EK. Technology-Based Alcohol Interventions in Primary Care: Systematic Review. *J Med Internet Res*. 2019;21(4):e10859. doi: 10.2196/10859 30958270 PMC6475823

[21] Boumparis N, Karyotaki E, Schaub MP, Cuijpers P, Riper H. Internet interventions for adult illicit substance users: a meta-analysis. *Addiction*. 2017;112(9):1521–1532. doi: 10.1111/add.13819 28295758 PMC5573910

[22] Boumparis N, Schulte MHJ, Riper H. Digital Mental Health for Alcohol and Substance Use Disorders. *Curr Treat Options Psych*. 2019;6: 352–66. doi: 10.1007/s40501-019-00190-y

[23] Chebli JL, Blaszczynski A, Gainsbury SM. Internet-Based Interventions for Addictive Behaviours: A Systematic Review. *J Gambl Stud*. 2016;32(4):1279–1304. doi: 10.1007/s10899-016-9599-5 27002522

[24] Tofighi B, Chemi C, Ruiz-Valcarcel J, Hein P, Hu L. Smartphone Apps Targeting Alcohol and Illicit Substance Use: Systematic Search in in Commercial App Stores and Critical Content Analysis. *JMIR Mhealth Uhealth*. 2019;7(4):e11831. doi: 10.2196/11831 31008713 PMC6658280

[25] Ferreri F, Bourla A, Mouchabac S, Karila L. e-Addictology: An Overview of New Technologies for Assessing and Intervening in Addictive Behaviors. *Front Psychiatry*. 2018;9:51. doi: 10.3389/fpsyt.2018.00051 29545756 PMC5837980

[26] Nesvåg S, McKay JR. Feasibility and Effects of Digital Interventions to Support People in Recovery From Substance Use Disorders: Systematic Review. *J Med Internet Res*. 2018;20(8):e255. doi: 10.2196/jmir.9873 30139724 PMC6127498

[27] Naslund JA, Aschbrenner KA, Araya R, Marsch LA, Unützer J, Patel V, et al. Digital technology for treating and preventing mental disorders in low-income and middle-income countries: a narrative review of the literature. *Lancet Psychiatry*. 2017;4(6):486–500. doi: 10.1016/S2215-0366(17)30096-2 28433615 PMC5523650

[28] Carter H, Araya R, Anjur K, Deng D, Naslund JA. The emergence of digital mental health in low-income and middle-income countries: A review of recent advances and implications for the treatment and prevention of mental disorders. *J Psychiatr Res*. 2021; 133:223–246. doi: 10.1016/j.jpsychires.2020.12.016 33360867 PMC8801979

[29] Murphy SM, Campbell AN, Ghitza UE, Kyle TL, Bailey GL, Nunes EV, et al. Cost-effectiveness of an internet-delivered treatment for substance abuse: Data from a multisite randomized controlled trial. *Drug Alcohol Depend*. 2016; 161:119–126. doi: 10.1016/j.drugalcdep.2016.01.021 26880594 PMC4792755

[30] Milward J, Day E, Wadsworth E, Strang J, Lynskey M. Mobile phone ownership, usage and readiness to use by patients in drug treatment. *Drug Alcohol Depend*. 2015; 146:111–115. doi: 10.1016/j.drugalcdep.2014.11.001 25468818

[31] McClure EA, Acquavita SP, Harding E, Stitzer ML. Utilization of communication technology by patients enrolled in substance abuse treatment. *Drug Alcohol Depend*. 2013;129(1–2):145–150. doi: 10.1016/j.drugalcdep.2012.10.003 23107600 PMC3568219

[32] Erbe D, Eichert HC, Riper H, Ebert DD. Blending Face-to-Face and Internet-Based Interventions for the Treatment of Mental Disorders in Adults: Systematic Review. *J Med Internet Res*. 2017;19(9):e306. doi: 10.2196/jmir.6588 28916506 PMC5622288

[33] Lin LA, Casteel D, Shigekawa E, Weyrich MS, Roby DH, McMenamin SB. Telemedicine-delivered treatment interventions for substance use disorders: A systematic review. *J Subst Abuse Treat*. 2019;101:38–49. doi: 10.1016/j.jsat.2019.03.007 31006553

[34] Lin LA, Fernandez AC, Bonar EE. Telehealth for Substance-Using Populations in the Age of Coronavirus Disease 2019: Recommendations to Enhance Adoption. *JAMA Psychiatry*. 2020;77(12):1209–1210. doi: 10.1001/jamapsychiatry.2020.1698 32609317 PMC8108064

[35] Bowen DJ, Kreuter M, Spring B, Cofta-Woerpel L, Linnan L, Weiner D, et al. How we design feasibility studies. *Am J Prev Med*. 2009;36(5):452–457. doi: 10.1016/j.amepre.2009.02.002 19362699 PMC2859314

[36] Sirriyeh R, Lawton R, Gardner P, Armitage G. Reviewing studies with diverse designs: the development and evaluation of a new tool. *J Eval Clin Pract*. 2012;18(4):746–752. doi: 10.1111/j.1365-2753.2011.01662.x 21410846

[37] Scoping Reviews: Quality Assessment. July 25, 2022. Available from: https://guides.lib.unc.edu/scoping-reviews/assessing-quality.

[38] Arksey H, O’Malley L. Scoping studies: towards a methodological framework. *Int J Soc Res Methodol*. 2005. 8:19–32. doi: 10.1080/1364557032000119616

[39] Levac D., Colquhoun H. & O’Brien K.K. Scoping studies: advancing the methodology. *Implementation Sci*. 2010;5:69. doi: 10.1186/1748-5908-5-69 20854677 PMC2954944

[40] Peters MD, Godfrey CM, Khalil H, McInerney P, Parker D, Soares CB. Guidance for conducting systematic scoping reviews. *Int J Evid Based Healthc*. 2015;13(3):141–146. doi: 10.1097/XEB.0000000000000050 26134548

[41] O’Connor D, Green S, Higgins JP. Defining the review question and developing criteria for including studies. In: Cochrane handbook for systematic reviews of interventions: Cochrane book series; 2008. p. 81–94. (cited 2022 August 30). Available from 10.1002/9780470712184.ch5

[42] Proctor E, Silmere H, Raghavan R, Hovmand P, Aarons G, Bunger A, et al. Outcomes for implementation research: conceptual distinctions, measurement challenges, and research agenda. *Adm Policy Ment Health*. 2011;38(2):65–76. doi: 10.1007/s10488-010-0319-7 20957426 PMC3068522

[43] Sekhon M, Cartwright M & Francis JJ. Acceptability of healthcare interventions: an overview of reviews and development of a theoretical framework. *BMC Health Serv Res*.2017;17(88). 10.1186/s12913-017-2031-8 PMC5267473 28126032

[44] Chen J, Qian M, Sun C, Lin M, Tang W. Clinical effectiveness of cognitive behavioural therapy on alcohol-dependent patients: an observation with the WeChat platform. *Gen Psychiatr*. 2019;32(5):e100087. doi: 10.1136/gpsych-2019-100087 31673676 PMC6802970

[45] Chen J, Ho E, Jiang Y, Whittaker R, Yang T, Bullen C. Mobile Social Network-Based Smoking Cessation Intervention for Chinese Male Smokers: Pilot Randomized Controlled Trial. *JMIR Mhealth Uhealth*. 2020;8(10):e17522. doi: 10.2196/17522 33095184 PMC7647814

[46] Ybarra ML, Holtrop JS, Bağci Bosi AT, Bilir N, Korchmaros JD, Salih Emri AK. Feasibility and acceptability of a text messaging-based smoking cessation program in Ankara, Turkey. *J Health Commun*. 2013;18(8):960–973. doi: 10.1080/10810730.2012.757399 23627304

[47] Biswas MR, Alzubaidi MS, Shah U, Abd-Alrazaq AA, Shah Z. A Scoping Review to Find Out Worldwide COVID-19 Vaccine Hesitancy and Its Underlying Determinants. *Vaccines (Basel)*. 2021;9(11):1243. doi: 10.3390/vaccines9111243 34835174 PMC8624792

[48] Liberati A, Altman DG, Tetzlaff J, Mulrow C, Gøtzsche PC, Ioannidis JP, et al. The PRISMA statement for reporting systematic reviews and meta-analyses of studies that evaluate healthcare interventions: explanation and elaboration. *BMJ*. 2009;339:b2700. doi: 10.1136/bmj.b2700 19622552 PMC2714672

[49] Ybarra M, Bağci Bosi AT, Korchmaros J, Emri S. A text messaging-based smoking cessation program for adult smokers: randomized controlled trial [published correction appears in J Med Internet Res. 2015;17(6):e125]. *J Med Internet Res*. 2012;14(6):e172. doi: 10.2196/jmir.2231 23271159 PMC3799568

[50] Önür ST, Uysal MA, İliaz S, et al. Does Short Message Service Increase Adherence to Smoking Cessation Clinic Appointments and Quitting Smoking? *Balkan Med J*. 2016;33(5):525–531. doi: 10.5152/balkanmedj.2016.151610 27761280 PMC5056655

[51] Akhu-Zaheya LM, Shiyab WY. The effect of short message system (SMS) reminder on adherence to a healthy diet, medication, and cessation of smoking among adult patients with cardiovascular diseases. *Int J Med Inform*. 2017;98:65–75. doi: 10.1016/j.ijmedinf.2016.12.003 28034414

[52] Borzekowski D.L.G., Chen J.C. Tobacco cues in India: An ecological momentary assessment. *Tob*. *Induced Dis*. 2016;14:16. doi: 10.1186/s12971-016-0081-z 27147939 PMC4855761

[53] Wu L, He Y, Jiang B, Zhang D, Tian H, Zuo F, et al. Very brief physician advice and supplemental proactive telephone calls to promote smoking reduction and cessation in Chinese male smokers with no intention to quit: a randomized trial. *Addiction*. 2017;112(11):2032–2040. doi: 10.1111/add.13908 28623848

[54] Yu S, Duan Z, Redmon PB, Eriksen MP, Koplan JP, Huang C. mHealth Intervention is Effective in Creating Smoke-Free Homes for Newborns: A Randomized Controlled Trial Study in China. *Sci Rep*. 2017;7(1):9276. doi: 10.1038/s41598-017-08922-x 28860461 PMC5578962

[55] Nădăşan V, Foley KL, Pénzes M, Paulik E, Mihăicuţă Ș, Ábrám Z, et al. The Short-term Effects of ASPIRA: A Web-based, Multimedia Smoking Prevention Program for Adolescents in Romania: A Cluster Randomized Trial. *Nicotine Tob Res*. 2017;19(8):908–915. doi: 10.1093/ntr/ntw308 27838661 PMC5896509

[56] Andrade AL, de Lacerda RB, Gomide HP, Ronzani TM, Sartes LM, Martins L, et al. Web-based self-help intervention reduces alcohol consumption in both heavy-drinking and dependent alcohol users: A pilot study. *Addict Behav*. 2016;63:63–71. doi: 10.1016/j.addbeh.2016.06.027 27424165

[57] Augustson E, Engelgau MM, Zhang S, Cai Y, Cher W, Li R, et al. Text to Quit China: An mHealth Smoking Cessation Trial. *Am J Health Promot*. 2017;31(3):217–225. doi: 10.4278/ajhp.140812-QUAN-399 26730560 PMC4935631

[58] Blitchtein-Winicki D, Zevallos K, Samolski MR, Requena D, Velarde C, Briceño P, et al. Feasibility and Acceptability of a Text Message-Based Smoking Cessation Program for Young Adults in Lima, Peru: Pilot Study. *JMIR Mhealth Uhealth*. 2017;5(8):e116. doi: 10.2196/mhealth.7532 28778850 PMC5562935

[59] Liao Y, Wu Q, Kelly BC, Zhang F, Tang YY, Wang Q, et al. Effectiveness of a text-messaging-based smoking cessation intervention ("Happy Quit") for smoking cessation in China: A randomized controlled trial. *PLoS Med*. 2018;15(12):e1002713. doi: 10.1371/journal.pmed.1002713 30562352 PMC6298640

[60] Liang D, Han H, Du J, Zhao M, Hser YI. A pilot study of a smartphone application supporting recovery from drug addiction. *J Subst Abuse Treat*. 2018; 88:51–58. doi: 10.1016/j.jsat.2018.02.006 29606226 PMC5884452

[61] Tran BX, Le XTT, Nguyen PN, Le Q, Mai HT, Nguyen H et al. Feasibility of e-Health Interventions on Smoking Cessation among Vietnamese Active Internet Users. *Int J Environ Res Public Health*. 2018;15(1):165. doi: 10.3390/ijerph15010165 29361694 PMC5800264

[62] Zhu Y, Jiang H, Su H, Zhong N, Li R, Li X, et al. A Newly Designed Mobile-Based Computerized Cognitive Addiction Therapy App for the Improvement of Cognition Impairments and Risk Decision Making in Methamphetamine Use Disorder: Randomized Controlled Trial. *JMIR Mhealth Uhealth*. 2018;6(6):e10292. doi: 10.2196/10292 29925497 PMC6031898

[63] Bernardes-Souza B, Patruz Ananias De Assis Pires F, Madeira GM, Felício Da Cunha Rodrigues T, Gatzka M, Heppt MV et al. Facial-Aging Mobile Apps for Smoking Prevention in Secondary Schools in Brazil: Appearance-Focused Interventional Study. *JMIR Public Health Surveill*. 2018;4(3):e10234. Published 2018 Jul 17. doi: 10.2196/10234 30021713 PMC6068381

[64] Sanchez ZM, Sanudo A. Web-based alcohol intervention for nightclub patrons: Opposite effects according to baseline alcohol use disorder classification. *Subst Abus*. 2018;39(3):361–370. doi: 10.1080/08897077.2018.1437586 29424680

[65] Tiburcio M, Lara MA, Martínez N, Fernández M, Aguilar A. Web-Based Intervention to Reduce Substance Abuse and Depression: A Three Arm Randomized Trial in Mexico. *Subst Use Misuse*. 2018;53(13):2220–2231. doi: 10.1080/10826084.2018.1467452 29768070

[66] Valente JY, Moreira TC, Ferigolo M, Barros HMT. Randomized clinical trial to change parental practices for drug use in a telehealth prevention program: a pilot study. *J Pediatr (Rio J)*. 2019;95(3):334–341. doi: 10.1016/j.jped.2018.02.004 29571681

[67] Han H, Zhang JY, Hser YI, Liang D, Li X, Wang SS, et al. Feasibility of a Mobile Phone App to Support Recovery From Addiction in China: Secondary Analysis of a Pilot Study. *JMIR Mhealth Uhealth*. 2018;6(2):e46. doi: 10.2196/mhealth.8388 29487040 PMC5849798

[68] Cupertino AP, Cartujano-Barrera F, Perales J, Formagini T, Rodríguez-Bolaños R, Ellerbeck EF, et al. "Vive Sin Tabaco… ¡Decídete!" Feasibility and Acceptability of an e-Health Smoking Cessation Informed Decision-Making Tool Integrated in Primary Healthcare in Mexico. *Telemed J E Health*. 2019;25(5):425–431. doi: 10.1089/tmj.2017.0299 30048208 PMC6916521

[69] Cupertino AP, Cartujano-Barrera F, Ramírez M, Rodríguez-Bolaños R, Thrasher JF, Pérez-Rubio G, et al. A Mobile Smoking Cessation Intervention for Mexico (Vive sin Tabaco… ¡Decídete!): Single-Arm Pilot Study. *JMIR Mhealth Uhealth*. 2019;7(4):e12482. doi: 10.2196/12482 31021326 PMC6658244

[70] Cruvinel E, Richter KP, Colugnati F, Ronzani TM. An Experimental Feasibility Study of a Hybrid Telephone Counseling/Text Messaging Intervention for Post-Discharge Cessation Support Among Hospitalized Smokers in Brazil. Nicotine Tob Res. 2019;21(12):1700–1705. doi: 10.1093/ntr/nty165 30137529 PMC6861823

[71] Durmaz S, Ergin I, Durusoy R, Hassoy H, Caliskan A,Okyay et al. WhatsApp embedded in routine service delivery for smoking cessation: effects on abstinence rates in a randomized controlled study. *BMC Public Health*. 2019;19: 387. doi: 10.1186/s12889-019-6727-z 30961557 PMC6454636

[72] Bedendo A, Ferri CP, de Souza AAL, Andrade ALM, Noto AR. Pragmatic randomized controlled trial of a web-based intervention for alcohol use among Brazilian college students: Motivation as a moderating effect. *Drug Alcohol Depend*. 2019;199:92–100. doi: 10.1016/j.drugalcdep.2019.02.021 31029880

[73] Bedendo A, McCambridge J, Gaume J, Souza AAL, Formigoni MLOS, Noto AR. Components evaluation of a web-based personalized normative feedback intervention for alcohol use among college students: a pragmatic randomized controlled trial with a dismantling design. *Addiction*. 2020;115(6):1063–1074. doi: 10.1111/add.14923 31785189

[74] Marzo RR, Bhattacharya S, Ravichandran S, Lakshmanan P, Jeffery VR, Moralitheran P, et al. Educating school students and gauging their perception about the harmful effects of smoking using a "Facial-Ageing App (mobile application):" An experience from Malaysia. *J Educ Health Promot*. 2019;8:250. Published 2019 Dec 31. doi: 10.4103/jehp.jehp_192_19 32002422 PMC6967115

[75] Lim JH, Ha Y. Effectiveness of a Workplace Smoking Cessation Program based on Self-determination Theory Using Individual Counseling and Tailored Text Messaging: A Pilot Study. *Asian Nurs Res (Korean Soc Nurs Sci)*. 2019;13(1):53–60. doi: 10.1016/j.anr.2019.01.004 30659928

[76] Nandyal M, Chandramouleeswaran S, Braganza D. Feasibility of mobile telephonic follow-up among patients with alcohol dependence syndrome. *Natl Med J India*. 2019;32(2):77–82. doi: 10.4103/0970-258X.275345 31939401

[77] Louwagie GM, Morojele N, Siddiqi K, Mdege ND, Tumbo J, Omole O, et al. Addressing tobacco smoking and drinking to improve TB treatment outcomes, in South Africa: a feasibility study of the ProLife program. [published correction appears in Transl Behav Med. 2022 May 26;12(5):721]. *Transl Behav Med*. 2020;10(6):1491–1503. doi: 10.1093/tbm/ibz100 31233146

[78] Nădăşan V, Ferencz L, Ábrám Z, Foley K. Predictors of High Program Exposure Among Adolescents Participating in a Smoking Prevention Intervention in Romania. *Tob Use Insights*. 2019;12:1179173X19845337. doi: 10.1177/1179173X19845337 31065218 PMC6487747

[79] Cupertino AP, Cartujano-Barrera F, Basile Colugnati FA, Batista Formagini TD, Garcia de Siqueira Galil A, Ferreira Carvalho Banhato E, et al. Web-based decision-making tool for smoking cessation (Pare de fumar conosco) among patients with chronic conditions in Brazil: one-arm feasibility study. *BMJ Health Care Inform*. 2020;27(1):e100063. doi: 10.1136/bmjhci-2019-100063 31915181 PMC7062353

[80] Harder VS, Musau AM, Musyimi CW, Ndetei DM, Mutiso VN. A randomized clinical trial of mobile phone motivational interviewing for alcohol use problems in Kenya. *Addiction*. 2020;115(6):1050–1060. doi: 10.1111/add.14903 31782966 PMC8353663

[81] Do VV, Spears CA, Van Minh H, et al. Perceptions About Mindfulness and Text Messaging for Smoking Cessation in Vietnam: Results from a Qualitative Study. *JMIR Mhealth Uhealth*. 2020;8(6):e17337. doi: 10.2196/17337 32442140 PMC7381024

[82] Cartujano-Barrera F, Sanderson Cox L, Arana-Chicas E, Ramírez M, Perales-Puchalt J, Valera P, et al. Feasibility and Acceptability of a Culturally- and Linguistically-Adapted Smoking Cessation Text Messaging Intervention for Latino Smokers. *Front Public Health*. 2020;8:269. doi: 10.3389/fpubh.2020.00269 32714891 PMC7344180

[83] Bertholet N., Cunningham J.A. Information technology and addiction science: promises and challenges. *Addict Sci Clin Pract*. 2021;16:7. doi: 10.1186/s13722-021-00216-y 33499925 PMC7836206

[84] Bandawar M, Narasimha VL, Chand P. Use of digital technology in addiction disorders. *Indian J Psychiatry*. 2018;60(Suppl 4):S534–S540. doi: 10.4103/psychiatry.IndianJPsychiatry_21_18 29540927 PMC5844168

[85] Acharibasam JW, Wynn R. Telemental Health in Low- and Middle-Income Countries: A Systematic Review. *Int J Telemed Appl*. 2018;2018:9602821. Published 2018 Nov 1. doi: 10.1155/2018/9602821 30519259 PMC6241375

[86] Internet Users By Country 2022-Statista. Available from: https://www.statista.com/statistics/262966/number-of-internet-users-in-selected-countries/

[87] Brazil- smartphone penetration rate 205-206- Statistica. Available from: https://www.statista.com/forecasts/625406/smartphone-user-penetration-in-brazil

[88] Drug and age-UNODC.Available from https://www.unodc.org/wdr2018/prelaunch/WDR18_Booklet_4_YOUTH.pdf

[89] WHO. Tobacco—World Health Organization. July 2021. Available from: https://www.who.int/news-room/fact-sheets/detail/tobacco.//Health effects of tobacco use-FDA food and drugs. https://www.fda.gov/tobacco-products/public-health-education/health-effects-tobacco-use

[90] Collins KM, Armenta RF, Cuevas-Mota J, Liu L, Strathdee SA, Garfein RS. Factors associated with patterns of mobile technology use among persons who inject drugs. *Subst Abus*. 2016;37(4):606–612. doi: 10.1080/08897077.2016.1176980 27092425 PMC5125293

[91] Marsch LA, Borodovsky JT. Digital health interventions for substance use disorders: The state of the science, in ASAM Principles of Addiction Medicine, 2018. Miller SC, et al., Editors. Lippincott Williams and Wilkins: Philadelphia, PA.

