# Supplementary figures and images for: Telehealth interventions for substance use disorders in low- and- middle income countries: A scoping review
Source: PLOS Digit Health. 2022 Nov 2;1(11):e0000125. doi: 10.1371/journal.pdig.0000125 (PMC9931245; doi:10.1371/journal.pdig.0000125)

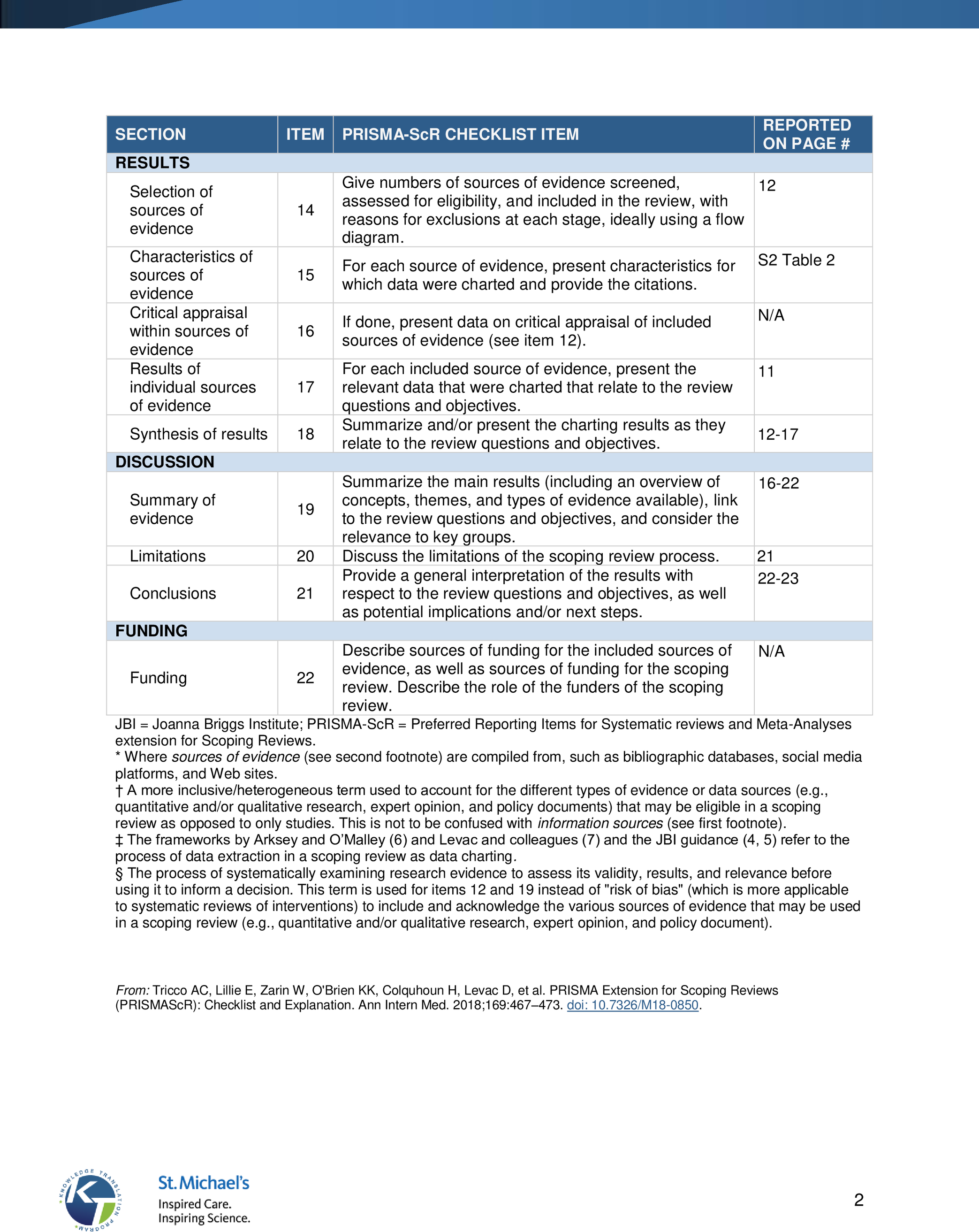

Supplement: S1 PRISMA Checklist — (TIF) [file pdig.0000125.s001.tif]

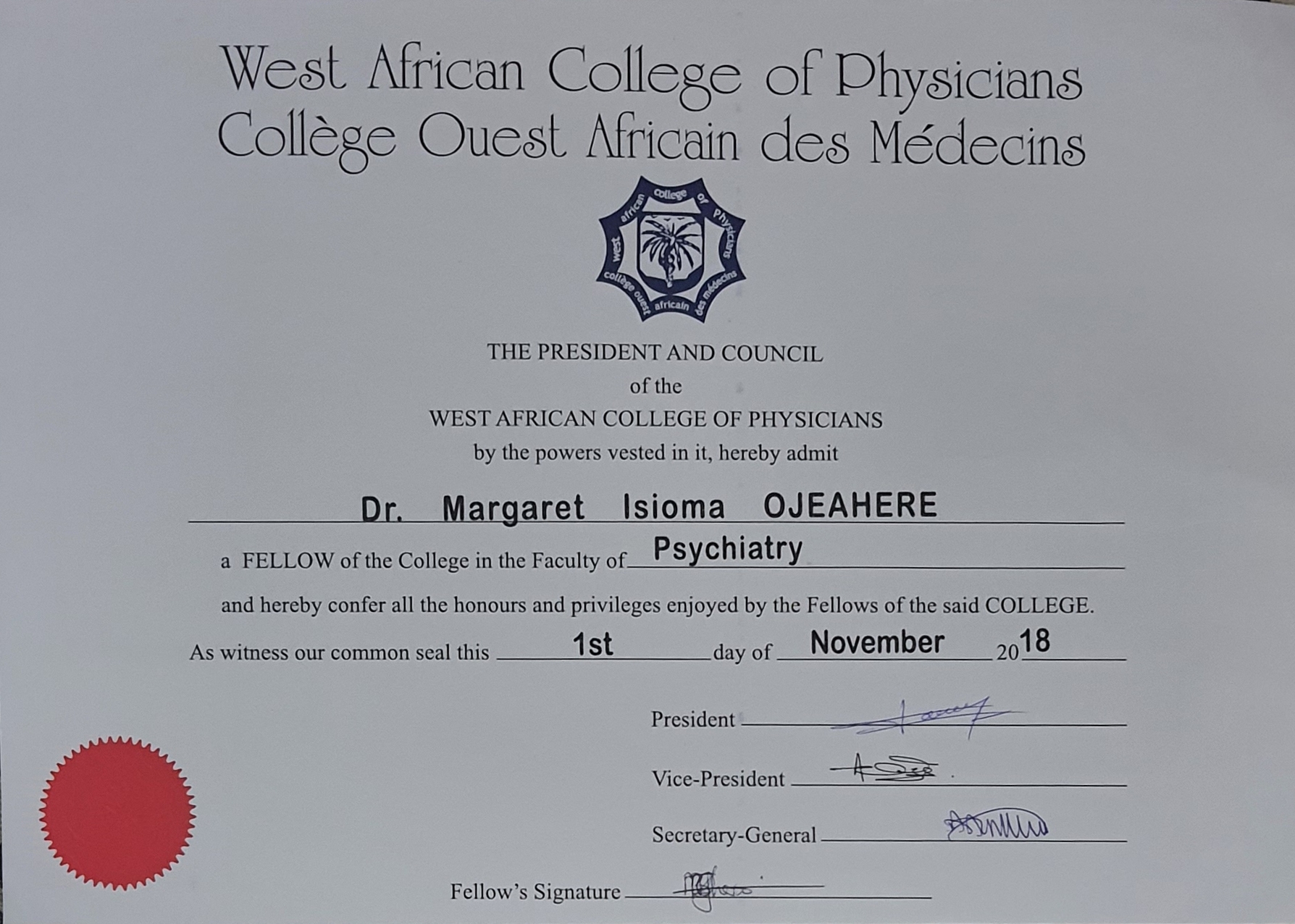

Supplement: S1 Data — (ZIP) [file pdig.0000125.s002.zip › CERTIFICATE OF QUALIFICATION AS A FELLOW OF WACP.jpg]
