# Supplementary material for: Telehealth interventions for substance use disorders in low- and- middle income countries: A scoping review
Source: PLOS Digit Health. 2022 Nov 2;1(11):e0000125. doi: 10.1371/journal.pdig.0000125 (PMC9931245; doi:10.1371/journal.pdig.0000125)
Supplement: S1 Data — (ZIP) [file pdig.0000125.s002.zip › Database searches /Web of Science [v.5.35] - Web of Science Core Collection Advanced Search.pdf]

Web of Science InCites Journal Citation Reports Essential Science Indicators EndNote Publons Kopernio Master Journal List Sign In Help English

Web of Science

Clarivate Analytics

Tools Searches and alerts Search History Marked List

Select a database Web of Science Core Collection

Basic Search Author Search BETA Cited Reference Search **Advanced Search**

Use field tags, Boolean operators, parentheses, and query sets to create your query. Results will appear in the Search History table at the bottom of the page.(Learn more about Advanced Search)

Example: TS=(nanotub* AND carbon) NOT AU=Smalley RE
#1 NOT #2 more examples | view the tutorial

Search

Restrict results by languages and document types:

All languages	All document types
English	Article
Afrikaans	Abstract of Published Item
Arabic	Art Exhibit Review

Booleans: AND, OR, NOT, SAME, NEAR

Field Tags:

**TS**= Topic	**SA**= Street Address
**TI**= Title	**CI**= City
**AU**= Author [Index]	**PS**= Province/State
**AI**= Author Identifiers	**CU**= Country/Region
**GP**= Group Author [Index]	**ZP**= Zip/Postal Code
**ED**= Editor	**FO**= Funding Agency
**SO**= Publication Name [Index]	**FG**= Grant Number
**DO**= DOI	**FT**= Funding Text
**PY**= Year Published	**SU**= Research Area
**CF**= Conference	**WC**= Web of Science Category
**AD**= Address	**IS**= ISSN/ISBN
**OG**= Organization-Enhanced [Index]	**UT**= Accession Number
**OO**= Organization	**PMID**= PubMed ID
**SG**= Suborganization	**ALL**= All Fields
**AB**= Abstract	
**AK**= Author Keywords	
**KP**= Keyword Plus ®	

Timespan

Custom year range 2000 to 2020

More settings

(To save these permanently, sign in or register.)

Web of Science Core Collection: Citation Indexes

- [x] Science Citation Index Expanded (SCI-EXPANDED) --1945-present
- [x] Social Sciences Citation Index (SSCI) --1956-present
- [x] Arts & Humanities Citation Index (A&HCI) --1975-present
- [x] Conference Proceedings Citation Index- Science (CPCI-S) --2010-present
- [x] Conference Proceedings Citation Index- Social Science & Humanities (CPCI-SSH) --2010-present
- [x] Book Citation Index– Science (BKCI-S) --2010-present
- [x] Book Citation Index– Social Sciences & Humanities (BKCI-SSH) --2010-present
- [x] Emerging Sources Citation Index (ESCI) --2015-present

Data last updated: 2020-09-29

Search History:

Set Results Save History / Create Alert Open Saved History Edit Sets Com

Set	Results		Edit Sets
# 5	108	#4 AND #3 AND #2 AND #1<br>*Indexes=SCI-EXPANDED, SSCI, A&HCI, CPCI-S, CPCI-SSH, BKCI-S, BKCI-SSH, ESCI Timespan=2000-2020*	Edit

# 4	2,480,241	TS=(Afghanistan OR Albania OR Algeria OR “American Samoa” OR Angola OR Argentina OR “Argentine Republic” OR Armenia OR Azerbaijan OR Bangladesh OR Belarus OR Byelarus OR Belorussia OR Belize OR Benin OR Bhutan OR Bolivia OR Bosnia OR Botswana OR Brazil OR Bulgaria OR Burma OR “Burkina Faso” OR Burundi OR “Cabo Verde” OR “Cape verde” OR Cambodia OR Cameroon OR “Central African Republic” OR Chad OR China OR Colombia OR Comoros OR Comores OR Comoro OR Congo OR “Costa Rica” OR “Côte d'Ivoire” OR Cuba OR Djibouti OR Dominica OR “Dominican Republic” OR Ecuador OR Egypt OR “El Salvador” OR Eritrea OR Ethiopia OR Fiji OR Gabon OR Gambia OR Gaza OR “Georgia Republic” OR Georgian OR Ghana OR Grenada OR Grenadines OR Guatemala OR Guinea OR “Guinea Bissau” OR Guyana OR Haiti OR Herzegovina OR Hercegovina OR Honduras OR India OR Indonesia OR Iran OR Iraq OR Jamaica OR Jordan OR Kazakhstan OR Kenya OR Kiribati OR Korea OR Kosovo OR Kyrgyz OR Kirghizia OR Kirghiz OR Kirgizstan OR Kyrgyzstan OR “Lao PDR” OR Laos OR Lebanon OR Lesotho OR Liberia OR Libya OR Macedonia OR Madagascar OR Malawi OR Malay OR Malaya OR Malaysia OR Maldives OR Mali OR “Marshall Islands” OR Mauritania OR Mauritius OR Mexico OR Micronesia OR Moldova OR Mongolia OR Montenegro OR Morocco OR Mozambique OR Myanmar OR Namibia OR Nauru OR Nepal OR Nicaragua OR Niger OR Nigeria OR Pakistan OR Palau OR Panama OR “Papua New Guinea” OR Paraguay OR Peru OR Philippines OR Phillippines OR Philipines OR Phillipines OR Principe OR Romania OR Rwanda OR Ruanda OR Samoa OR “Sao Tome” OR Senegal OR Serbia OR “Sierra Leone” OR “Solomon Islands” OR Somalia OR “South Africa” OR “South Sudan” OR “Sri Lanka” OR “St Lucia” OR “St Vincent” OR Sudan OR Surinam OR Suriname OR Swaziland OR Syria OR “Syrian Arab Republic” OR Tajikistan OR Tadzhikistan OR Tadjikistan OR Tadzhik OR Tanzania OR Thailand OR Timor OR Togo OR Tonga OR Tunisia OR Turkey OR Turkmen OR Turkmenistan OR Tuvalu OR Uganda OR Ukraine OR Uzbek OR Uzbekistan OR Vanuatu OR Venezuela OR Vietnam OR “West Bank” OR Yemen OR Zambia OR Zimbabwe)<br>*Indexes=SCI-EXPANDED, SSCI, A&HCI, CPCI-S, CPCI-SSH, BKCI-S, BKCI-SSH, ESCI Timespan=2000-2020*	Edit
# 3	1,195,240	TS=(Feasibility OR effectiveness)<br>*Indexes=SCI-EXPANDED, SSCI, A&HCI, CPCI-S, CPCI-SSH, BKCI-S, BKCI-SSH, ESCI Timespan=2000-2020*	Edit
# 2	586,265	TS=(“Substance use” OR “Substance use disorder” OR “substance abuse” OR “substance dependence” OR addiction OR addict OR “alcohol use disorder” OR “alcohol abuse” OR “alcohol dependence” OR “alcohol addiction” OR tobacco OR cigarette OR smoking OR nicotine OR cannabis OR marijuana OR bhang OR Khat OR shisha OR heroin OR opioid OR “injecting drug use” OR “people with injecting drug use” OR PWID OR cocaine OR amphetamine OR methamphetamine)<br>*Indexes=SCI-EXPANDED, SSCI, A&HCI, CPCI-S, CPCI-SSH, BKCI-S, BKCI-SSH, ESCI Timespan=2000-2020*	Edit
# 1	580,595	TS=(telehealth OR telepsychiatry OR telemedicine OR teleconsultation OR “mobile health” OR mhealth OR “mobile phone” OR web OR “video conferencing” OR SMS OR “short message” OR internet)<br>*Indexes=SCI-EXPANDED, SSCI, A&HCI, CPCI-S, CPCI-SSH, BKCI-S, BKCI-SSH, ESCI Timespan=2000-2020*	Edit

○ A

C

University of KwaZulu Natal

Clarivate Analytics

Read: How to save hundreds of hours and fly through your next research paper

Clarivate
Accelerating innovation

© 2020 Clarivate Copyright notice Terms of use Privacy statement Cookie policy

Sign up for the Web of Science newsletter Follow us
