# Supplementary material for: Telehealth interventions for substance use disorders in low- and- middle income countries: A scoping review
Source: PLOS Digit Health. 2022 Nov 2;1(11):e0000125. doi: 10.1371/journal.pdig.0000125 (PMC9931245; doi:10.1371/journal.pdig.0000125)
Supplement: S1 Data — (ZIP) [file pdig.0000125.s002.zip › Database searches /Print Search History_ PSYCHINFO.pdf]

Wednesday, September 30, 2020 6:20:08 PM

#	Query	Limiters/Expanders	Last Run Via	Results
S1	((telehealth OR telepsychiatry OR telemedicine OR teleconsultation OR “mobile health” OR mhealth OR “mobile phone” OR web OR “video conferencing” OR SMS OR “short message” OR internet)) AND ((“Substance use” OR “Substance use disorder” OR “substance abuse” OR “substance dependence” OR addiction OR addict OR “alcohol use disorder” OR “alcohol abuse” OR “alcohol dependence” OR “alcohol addiction” OR tobacco OR cigarette OR smoking OR nicotine OR cannabis OR marijuana OR bhang OR Khat OR shisha OR heroin OR opioid OR “injecting drug use” OR “people with injecting drug use” OR PWID OR cocaine OR amphetamine OR methamphetamine)) AND (Feasibility OR effectiveness) AND ((Afghanistan OR Albania OR Algeria OR “American Samoa” OR Angola OR Argentina OR “Argentine Republic” OR Armenia OR Azerbaijan OR Bangladesh OR Belarus	Limiters - Publication Year: 2000-2020; Published Date: 20000101-20201031; English Expanders - Apply equivalent subjects Search modes - Boolean/Phrase	Interface - EBSCOhost Research Databases Search Screen - Advanced Search Database - APA PsycInfo	128

OR Byelarus OR
Belorussia OR Belize OR
Benin OR Bhutan OR
Bolivia OR Bosnia OR
Botswana OR Brazil OR
Bulgaria OR Burma OR
“Burkina Faso” OR
Burundi OR “Cabo Verde”
OR “Cape verde” OR
Cambodia OR Cameroon
OR “Central African
Republic” OR Chad OR
China OR Colombia OR
Comoros OR Comores
OR Comoro OR Congo
OR “Costa Rica” OR
“Côte d'Ivoire” OR Cuba
OR Djibouti OR Dominica
OR “Dominican Republic”
OR Ecuador OR Egypt
OR “El Salvador” OR
Eritrea OR Ethiopia OR
Fiji OR Gabon OR
Gambia OR Gaza OR
“Georgia Republic” OR
Georgian OR Ghana OR
Grenada OR Grenadines
OR Guatemala OR
Guinea OR “Guinea
Bissau” OR Guyana OR
Haiti OR Herzegovina OR
Hercegovina OR
Honduras OR India OR
Indonesia OR Iran OR
Iraq OR Jamaica OR
Jordan OR Kazakhstan
OR Kenya OR Kiribati OR
Korea OR Kosovo OR
Kyrgyz OR Kirghizia OR
Kirghiz OR Kirgizstan OR
Kyrgyzstan OR “Lao PDR”
OR Laos OR Lebanon OR
Lesotho OR Liberia OR
Libya OR Macedonia OR
Madagascar OR Malawi
OR Malay OR Malaya OR

Malaysia OR Maldives OR Mali OR “Marshall Islands” OR Mauritania OR Mauritius OR Mexico OR Micronesia OR Moldova OR Mongolia OR Montenegro OR Morocco OR Mozambique OR Myanmar OR Namibia OR Nauru OR Nepal OR Nicaragua OR Niger OR Nigeria OR Pakistan OR Palau OR Panama OR “Papua New Guinea” OR Paraguay OR Peru OR Philippines OR Phillippines OR Philipines OR Phillipines OR Principe OR Romania OR Rwanda OR Ruanda OR Samoa OR “Sao Tome” OR Senegal OR Serbia OR “Sierra Leone” OR “Solomon Islands” OR Somalia OR ��South Africa” OR “South Sudan” OR “Sri Lanka” OR “St Lucia” OR “St Vincent” OR Sudan OR Surinam OR Suriname OR Swaziland OR Syria OR “Syrian Arab Republic” OR Tajikistan OR Tadzhikistan OR Tadjikistan OR Tadzhik OR Tanzania OR Thailand OR Timor OR Togo OR Tonga OR Tunisia OR Turkey OR Turkmen OR Turkmenistan OR Tuvalu OR Uganda OR Ukraine OR Uzbek OR Uzbekistan OR Vanuatu OR Venezuela OR Vietnam OR “West Bank” OR Yemen OR Zambia OR Zimbabwe))
