# Supplementary material for: Telehealth interventions for substance use disorders in low- and- middle income countries: A scoping review
Source: PLOS Digit Health. 2022 Nov 2;1(11):e0000125. doi: 10.1371/journal.pdig.0000125 (PMC9931245; doi:10.1371/journal.pdig.0000125)
Supplement: S1 Table — (DOCX) [file pdig.0000125.s003.docx]

S1 Table: Search String

| telehealth OR telepsychiatry OR telemedicine OR teleconsultation OR mobile health OR mhealth OR mobile phone OR web OR video conferencing OR SMS OR short message OR internet | AND | Substance use OR Substance use disorder OR substance abuse OR substance dependence OR addiction OR addict OR alcohol use disorder OR alcohol abuse OR alcohol dependence OR alcohol addiction OR tobacco OR cigarette OR smoking OR nicotine OR cannabis OR marijuana OR bhang OR Khat OR shisha OR heroin OR opioid OR injecting drug use OR people with injecting drug use OR PWID OR cocaine OR amphetamine OR methamphetamine | AND | Feasibility OR effectiveness | AND | (Afghanistan OR Albania OR Algeria OR “American Samoa”  OR Angola  OR Argentina  OR “Argentine Republic”  OR Armenia  OR Azerbaijan  OR Bangladesh  OR Belarus  OR Byelarus  OR Belorussia  OR Belize  OR Benin  OR Bhutan  OR Bolivia  OR Bosnia  OR Botswana  OR Brazil  OR Bulgaria  OR Burma  OR “Burkina Faso”  OR Burundi  OR “Cabo Verde”  OR “Cape verde”  OR Cambodia  OR Cameroon  OR “Central African Republic”  OR Chad  OR China  OR Colombia  OR Comoros  OR Comores  OR Comoro  OR Congo  OR “Costa Rica”  OR “Côte d'Ivoire”  OR Cuba  OR Djibouti  OR Dominica  OR “Dominican Republic”  OR Ecuador  OR Egypt  OR “El Salvador”  OR Eritrea  OR Ethiopia  OR Fiji  OR Gabon  OR Gambia  OR Gaza  OR “Georgia Republic”  OR Georgian  OR Ghana  OR Grenada  OR Grenadines  OR Guatemala  OR Guinea  OR “Guinea Bissau”  OR Guyana  OR Haiti  OR Herzegovina  OR Hercegovina  OR Honduras  OR India  OR Indonesia  OR Iran  OR Iraq  OR Jamaica  OR Jordan  OR Kazakhstan  OR Kenya  OR Kiribati  OR Korea  OR Kosovo  OR Kyrgyz  OR Kirghizia  OR Kirghiz  OR Kirgizstan  OR Kyrgyzstan  OR “Lao PDR”  OR Laos  OR Lebanon  OR Lesotho  OR Liberia  OR Libya  OR Macedonia  OR Madagascar  OR Malawi  OR Malay  OR Malaya  OR Malaysia  OR Maldives  OR Mali  OR “Marshall Islands”  OR Mauritania  OR Mauritius  OR Mexico  OR Micronesia  OR Moldova  OR Mongolia  OR Montenegro  OR Morocco  OR Mozambique  OR Myanmar  OR Namibia  OR Nauru  OR Nepal  OR Nicaragua  OR Niger  OR Nigeria   OR  Pakistan   OR  Palau  OR Panama  OR “Papua New Guinea”  OR  Paraguay  OR Peru   OR  Philippines  OR Phillippines  OR Philipines  OR Phillipines  OR Principe  OR Romania  OR Rwanda  OR Ruanda  OR Samoa  OR “Sao Tome”  OR Senegal  OR Serbia  OR “Sierra Leone”  OR “Solomon Islands”  OR Somalia  OR  “South Africa”  OR “South Sudan”  OR “Sri Lanka”  OR “St Lucia”  OR “St Vincent”  OR Sudan  OR Surinam  OR Suriname  OR Swaziland  OR Syria  OR “Syrian Arab Republic”  OR Tajikistan  OR Tadzhikistan  OR Tadjikistan  OR Tadzhik  OR Tanzania  OR Thailand  OR Timor  OR Togo  OR Tonga  OR Tunisia  OR Turkey  OR Turkmen  OR Turkmenistan  OR Tuvalu  OR Uganda  OR Ukraine  OR Uzbek  OR Uzbekistan  OR Vanuatu  OR Venezuela  OR Vietnam  OR “West Bank”  OR Yemen  OR Zambia  OR Zimbabwe ) |
| --- | --- | --- | --- | --- | --- | --- |
