# Supplementary material for: Telehealth interventions for substance use disorders in low- and- middle income countries: A scoping review
Source: PLOS Digit Health. 2022 Nov 2;1(11):e0000125. doi: 10.1371/journal.pdig.0000125 (PMC9931245; doi:10.1371/journal.pdig.0000125)
Supplement: S2 Table — (DOCX) [file pdig.0000125.s004.docx]

**S2 Table: Tables of selected articles showing telehealth intervention for Substance Use Disorders (SUDs) for Low- and Middle-Income Countries (LMICs)**

| **Author/ year** | **Country** | **Sample characteristic** | **Treatment modality**  **Substance targeted** | **Intervention delivery** | **Substance related outcome**  **(effectiveness/efficacy)** | **Feasibility /acceptability outcomes** | **Other outcomes** |
| --- | --- | --- | --- | --- | --- | --- | --- |
| Cupertino 2020 | Brazil | **Setting:**  Two healthcare centers  **Study population:** Individuals with chronic illness  **Sample size:**85  **Mean age (SD):** 54.2 | **Modality:**  Web-based program  (Smoking cessation tool name: Pare de fumar conosco)  **Substance targeted**  Tobacco | **Intervention group:** web-based intervention that used video, audio and animations  Lasted 15-25minutes  **Control group:**  None  **Delivered by:** self. but report generated after intervention to give to healthcare providers  **Number of sessions:** 1  **Follow up duration:**12 weeks | 7-days point prevalence abstinence at week 12:  9.4% (n=8) | **Recruitment:** 181 out of the 217 screened were eligible, but 85 agreed to participate  **Adherence/retention:** 86.4% reached on follow up.  12.9% could not be contacted later  35.2% completed 12 weeks  **Acceptability/ satisfaction**  Potential to deliver cessation treatment to hard to reach, socioeconomically disadvantaged populations and linkage to other counselling resources | 95.3% set a quit date after using program  Interest in using pharmacotherapy increased from 22.4% to 82.4% (p<0.0001)  Interest in attending group counselling increased 21.2% to 85.9%  Confidence in quitting smoking increased (p=0.02)  Interest in quitting smoking increased but not significant |
| Cupertino 2019 | Mexico | **Setting:**  Urban primary healthcare clinic  **Study population: S**mokers planning to quit in 30 days  **Sample size:** 40  **Mean age (SD):** 36 (10.7) | **Modality:**  Mobile intervention  (Program name:Vive sinTabaco *i* Decidete*!*)  **Substance targeted**  Tobacco | **Intervention group:**  Tablet-based software that collected data on smoking and generated personalized plan.  A three-component intervention.3 levels of message interactivity: pre-scheduled standard, keyword triggered standard message and counsellor personalized message Individualized text messages along the 12 weeks.  Pharmacotherapy when indicated  **Control group:**  None  **Delivered by:** counsellor trained in tobacco cessation  **Number of sessions:** One 10-15 minutes session and text messages for 12 weeks  **Follow up duration:**12 weeks | Biochemically verified abstinence at 12 weeks: 40% | **Recruitment:** 40 enrolled out of 72 eligible  Average of 180 automated messages over 12 weeks.  Average 21 messages per participant  **Interaction level:**  None-7%  Low-40%  Medium- 37%  High- 10%  Interaction high at beginning and near quit date and reduced progressively with few spikes on days of assessment.  **Adherence/retention:**  None disenrolled.  87.5% follow up at 12 weeks  **Acceptability/ satisfaction**  85.7% extremely satisfied or very satisfied.  48.5% reported inability to send message at some point | **Relapse:** 37% notified program of relapse.  17% set a new quit date |
| Cupertino 2019 | Mexico | **Setting:** Two primary health care clinics  **Study population:**  Federal workers  **Sample size:** 162  **Mean age (SD):** 18.87(15.13) | **Modality:** Web based program accessed via tablet computer  Telephone calls  (Program name:Vive sinTabaco *i* Decidete*!*)    **Substance targeted**  Tobacco | **Intervention group:** Tablet computer-based intervention that developed an individual quit plan based on data collected.  Gave information on tobacco cessation using video and audio features.  Lasted 15-20 minutes  **Control group:**  None  **Delivered by:** Self-reported. The records were shared with healthcare providers after intervention  **Number of sessions:** 1  **Follow up duration:**3 months | Smoking abstinence:  7-day point prevalence at 3 months: 19.1% on intention to treat analysis and 23.5% on per protocol analysis  Among continuing smokers, reduced average number of cigarettes per day at 3 months (9.6 to 3.5, p<0.001) | **Recruitment** 162 out of eligible 164 were  recruited from waiting room by research assistant.  **Adherence/retention:**  3 months follow up: 81.5%  **Acceptability/ satisfaction**  95.4% would recommend program to others  73.5% found tool helpful | **Interest in quitting:** 96.3%  Set a quit date with 3 months: 88.9%  Interest in using pharmacotherapy and group counselling: 83.3% and 93.2% respectively  Participation in group counselling: 19.7%  Reasons given by those not setting quit date: competing mental health issues, lack of information on tool, not enough clarity from video  **Open-ended comments on the program:**  Program motivated smokers to quit, helped them identify a quit date; gave new information; innovative program.  Limitations cited: absence of a comparison group, exclusion of low intensity smokers, non-use of biomarkers, difficulty with Wi-Fi connection,  problem with follow up |
| Cruvinel 2019 | Brazil | **Setting:** Post discharge treatment facility  **Study population:**  hospitalized smokers  **Sample size**: 66 (44 intervention, 22 control)  **Mean age (SD): 47.7 (11.5)** | **Modality:**  Hybrid: telephone call and text message  **Substance targeted**  Tobacco | **Intervention group:**  Single telephone call during first week, lasting 15-30 minutes  2 text messages per day for  8 days for those not ready to quit and 15 days for those ready to quit  **Control group:**  Standard treatment comprising education materials and brief intervention  **Delivered by:**  Counsellors trained in tobacco treatment  **Number of sessions:** 1 phone call  8-15 days  **Follow up duration:**  1 and 3 months | **Abstinence**  At one month, higher values were found in intervention but no significant difference (25% versus 9%, p=0.13)  At 3 months – higher in the treatment group (31.8%, versus 9.1%, p=0.04)  Biochemically verified abstinence, higher in treatment group but no statistical difference (20.5% versus 4.5%, p=0.09)  Reduced number of cigarettes per day among those with continued smoking at 1 and 3 months but no statistical difference | **Recruitment:**  Recruited by study staff during hospitalization. 96 were screened and 30 excluded.  **Adherence/retention:**Among the intervention group, 3 did not receive allocated intervention and 1 for the control group (phones numbers were disconnected).  **Feasibility:**  At one month-  Average telephone counselling 15 minutes  1186 messages sent to 41 participants  77.9% of messages received  97.6% maintained same number  **Acceptability/ satisfaction**  80.4% found text message as “helpful”  80.5% reported number of messages as “enough”  95.1 found call to be “just the right length”. | Some limitations include manually sent SMS, fewer messages sent to participants not ready to quit, follow up at 3 months, insufficient power to definitively test the interventions. |
| Liao 2018 | China | **Setting:**  Multiple setting (across 30 cities and provinces)  **Study population:**  **Sample size:** 1369  (HFM 674, LFM 284, control 411)  **Mean age (SD):** 38.1(9.7) | **Modality:**  Text message  (Happy Quit Program)  **Substance targeted**  Tobacco | **Intervention group:**  High frequency message (HFM): 3-5 messages per day before quit day and after quit day for 12 weeks. Then 3-5 per week for next 12 weeks follow up.  Low frequency message (LFM): 3-5 messages per week before quit day and after quit day for 12 weeks. Then 1-2 per week for next 12 weeks follow up.  **Control group:**  1 text message per week to thank and remind participants of follow up. Given the booklet at end of study  **Delivered by:** Self. However, report generated after intervention to give to healthcare providers  **Number of sessions:** varied depending on treatment arm as above  **Follow up duration:** monthly up to 24 weeks | The 7-day point quit rate from week 1 to 24 ranges from approximately 10% to over 26% with intervention and less than 4% to almost 12% without intervention.  Biochemically verified abstinence at 24 weeks:  Higher in both LFM versus control (6.5% vs 1.9%, p<0.001) and  LFM versus control 6.0% vs 1.9%, p=0.002  No difference between HFM and LFM (p=0.75)  Self-reported continuous abstinence at week 12 and 24  All significantly higher in LFM and HFM except for LFM at 8 weeks  Less cigarette use among those who continued smoking from week 1-24 for the 2 intervention groups | **Recruitment:**  Advertisement was done via radio, billboards, newspaper, online platforms, hospital and pharmacies.  Screening for eligibility and baseline data collection was done via telephone by study assistants.  1144 were ineligible out of the 2561 assessed. 1417 completed the baseline assessment but 48 dropped out.  **Adherence/retention:** 24 weeks retention: lower for LFM (HFM 83.2%, LFM 74.6%, Control 87.1% p<0.001)  No difference between HFM and control (p=0.087)  **Acceptability/ satisfaction**  Not clearly stated but suggestive of a successful program and recommended for use | The large sample size, strength, the combination of self-report and use of biomarkers were strengths. Limitations Enrollment did not meet target sample as indicated in the protocol: 1369 enrolled out of target 2000, small number of quitters, |
| Durmaz 2019 | Turkey | **Setting:**  Smoking cessation outpatient clinic  **Study population:** Smokers who sought help to stop smoking  **Sample size:** 132 (44 intervention, 88 control)  **Mean age (SD): 39.3 (12.1)** | **Modality:**  WhatsApp messages  **Substance targeted**  Tobacco | **Intervention group:**  WhatsApp message as follows: before quit day 7 messages per day  After quit day:  First month: daily message Second month: message every other day  Third month: message every 3 days  Messages were covering different topics  **Control group:**  Usual care: counseling and motivational interviewing. 45- 60 minutes session.  **Delivered by:** 5 physicians trained in tobacco cessation  **Number of sessions:** One session for the control group  Varied frequency of message for the intervention group  3 months treatment duration  **Follow up duration:** first and second week, 1st, 3rd and 6th month | **Abstinence**  At month 1:  Abstinence was higher for the intervention compared to the control group (OR 2.85, 95%CI 1.33-6.14)  Success to quit was higher in intervention compared to the control group (OR 3.51, 95% CI 1.30-9.44)  Month 3:  Higher quitting rate in intervention group (OR 2.34, 95%CI 1.07-5.09)  Higher quitting success in men (OR 2.93 ,955 CI 1.31-6.57)  **Month 6:**  High success in intervention group 2.31 95%CI 1.03-5.16) | **Recruitment:** 19 were excluded out of 151 screened. The process was done by a researcher.  **Engagement:**  4.5% of participants in intervention group refused to receive message after the first month  **Adherence/retention:**  Intervention group: 100% follow up at month 1 and 3,  2.3%lost to follow up at month 6  **Control:** retention at 1^st^ month was 97.7% and 95.5% for month 3 and 6 follow up  **Acceptability/ satisfaction**  Not reported | Factors associated with abstinence/ quitting-  Quitting lower in unemployed (OR=0.08, 95%CI 0.01-0.66)  High depression score associated with lower abstinence rate at month 1 and 3 (OR=0.89, 95%CI 0.80-0.99 and OR=0.88 95%CI 0.78-0.99, respectively  Increased in quit attempts associated with increase in abstinence at month 1  Older age associated with quit success at month 6 (1.04, 95%CI, 1.01-1.8)  No difference at month one and difference in 3rd month. Higher success of continuity in medication in the intervention group. |
| Goldenhersh 2020 | Argentina | **Setting:**  General population  **Study population:**  Volunteers who smoked  **Sample size:**120 (60 in treatment group and 60 in control group)  **Mean age (SD):** 43.25 (10.06) | **Modality:**  Mobile App  **Substance targeted**  Tobacco | **Intervention group:**  A 21-day interactive phase comprising daily of  2 main activities made available only after completing activities of the previous day  Activities in the app comprised: practice session formal mindfulness; virtual reality mindfulness; daily self-report; peer to peer. Support; mind connect support (prompts to encourage engagement with the program)  **Control group:**  None  **Delivered by:** Self report. However, a report generated after intervention was given to healthcare providers  **Number of sessions:**  2 per day for 21 days  **Follow up duration:**90 days | Cigarette consumption: Decreased in treatment group in the 3^rd^ week and post intervention (p<0.001)  Abstinence  Post intervention  30% in those fully adherent and 21% of those regularly adherent  At 90 days follow up 39% versus 33% | **Recruitment:** 1080 recruited via Adverts and TV using a VR cardboard headset. 150 were eligible but 30 did not confirm participation  **Engagement:**  Participants were contacted if had not used the system for 2 days  92% contacted by SMS once  65% contacted by SMS twice  53% contacted by SMS thrice  34% contacted by SMS and phone calls  Participation in chat room: low only 13 participants commented  **Adherence:**  For TG only  93% completed 21-day program  41% fully adherent (used system daily for 21 days)  59% regularly adherence (completed program in more than 21 days). TG reported a 33% sustained abstinence at 90 days compared to 20% in CG | Craving: reduction in self-reported craving over time in the treatment group (p=0.0005)  Readiness to quit: higher in treatment group post intervention (p=0.005)  Readiness to quit at baseline was associated with adherence at 90 days  Mindfulness  FFMQ scores lower than baseline at post intervention p=0.002 and 90 day follow up p<0.001)  Short follow up assessment time and non-identical follow up assessment time between groups |
| Liang 2018 | China | **Setting:** Three Methadone Maintenance Treatment (MMT) clinic  **Study population:** Adults who had used heroine or other psychoactive substance in past 30 days (and possessed a smartphone)  **Sample size:**  75 (50 intervention, 25 control)  **Mean age (SD):** 41.6 (8.0) | **Modality:**  Mobile App  (S-Health)  **Substance targeted**  Multiple substance;  Heroin, cocaine, methamphetamine, marijuana, MDMA | **Intervention group:**  Daily surveys on craving, effects, triggers, response to triggers and social contact.  Message tailored according to emotional group  **Control group:**  Text message on HIV prevention and other educational material  **Delivered by:** self. but report generated after intervention to give to healthcare providers  **Number of sessions:** daily for 4 weeks  **Follow up duration:**4 weeks | Drug use:  Less drug use in both groups during study period but no difference (p=0.08)  Intervention group less likely to have positive urine test and report drug use, but difference not statistically significant (p=0.49, and p=0.13, respectively) | **Recruitment:** was done from MMT clinics and network of social workers in Shanghai  **Engagement:**  Mean daily surveys 13.8, median 13.5  Number submitting daily surveys ranged from 35.4% to 66.7% and 10% did not submit any daily response.  **Adherence/retention:**  Only 5.3% lost to follow up  **Acceptability/ satisfaction**  most reported the intervention as easy to use and understand  53.3% (70.8% of treatment group) able to remember substance use behavior in pat week  More of those in the intervention group preferred to answer questions on phone versus face-face (46.8% and 36.2%)  No difference in rating among the groups. | Limited comparability between urine test and self-reported TLFB result and possible potential bias away from the null hypothesis were some limitations |
| Harder 2020 | Kenya | **Setting:** Primary health center  **Study population:** Adults who screened for alcohol use problems  **Sample size:**300  Immediate mobile MI=89  MI in person;65  Delayed  mobile MI=76  **Mean age/ age range (SD):**  38 (11.7) | Alcohol | **Intervention:** mobile motivational interview (MI)  The MI was one session given immediately after enrollment. Reassessment done after one month.  **Control group:**  1.a one-month waitlist followed by delayed mobile MI.  2. In-person MI group that received MI face-to-face.  **Modality:** mobile phone call  **Delivered by:** clinicians with Masters degree in nursing, doctoral degree in clinical psychology, or a medical degree  And trained on MI.  **Number of sessions:** one (which lasted 30 minutes)  **Follow up duration:** one month and 6 months | **Average drinking scores:**  **At month 1**  Those in the waitlist control group had an AUDIT-C score that was nearly three points higher than those in the intervention group (*P*<0005), controlling for baseline AUDIT-C scores.  No difference between in-person MI and mobile MI.  At 6 months.  The results for comparison between groups were inconclusive (p=0.34) | **Engagement:**  Challenges identified; disconnected mobile numbers, phones not charged or participants declining to pick calls  **Adherence/retention:**  Loss to follow-up at 1 month was 7%, 7% and 3%; and at 6 months 11%, 12% and 19% in the waiting list control, immediate mobile MI and in-person MI groups respectively.  **Acceptability/ satisfaction:**   not assessed. | None |
| Tran 2018 | Vietnam | **Setting:** Several universities and high schools in Vietnam  **Study population:** youths aged 15-25 years. in universities and high schools  **Sample size:**1082  **Mean age:** Not indicated **Age range:**  15-25 | **Modality**  Smartphone application  **Substance targeted:** Tobacco | **Intervention group:** NA | NA | **Acceptability:** Only 26.8% of the individuals indicated that they were willing to utilize a smartphone application to assist them in quitting. | Mean daily hours of using internet per day was 3.5  34.9% found health information on internet as useful or very useful.  Factors associated with willingness to pay for smartphone app to help in smoking cessation were having trust on health information on internet while those who had never shared health information on internet had reduced willingness. |
| Wu 2017 | China | **Setting:** Endocrinology and acupuncture  outpatient clinics of a general hospital  **Study population:** Male patients attending the  clinics  **Sample size:** 369 (intervention group-181 Control group-188**)**  **Mean age/ age range:** 40.4 years (range: 19 to 80 years). | **Modality:**  Telephone, face to face  **Substance targeted:** Tobacco | **Intervention:** Smoking reduction intervention group (SRI)  Received brief in-person session at baseline and call at 1 week and month 1,3,6, and 12 from baseline.  **Control group:** Exercise- and diet-advice group (EDA)  Received brief face to face session with advice on exercise and diet at baseline and at follow up,  **Delivered by:** Trained counselor  **Number of sessions:** 6 (1 face to face session and 5 telephone calls)  Lasting for about 1 minute  **Follow up duration:** 1,3,6 and 12 months | The 7-day point prevalence rate was slightly higher in the SRI than the EDA at each follow up interview  No statistical difference between the groups on the self-reported prolonged abstinence rate at 6-month and  at 12-month follow-up.  Biochemically validated quit rates at 12 months was 6.1% and 2.1% in SRI and EDA respectively, p=0.07 | **Recruitment**:  820 recruited but only 369 completed follow up  **Adherence/retention:**  45% completed treatment.  Participation rate for biochemically validated abstinence was 43.2% (45.8% for SRI and 38.5% for EDA)  **Acceptability/ satisfaction:** the study suggests that physicians face to face intervention encouraged retention and feasibility | None |
| Ybarra 2012 | Turkey | **Setting:** community members  **Study population:** adult daily smokers seriously thinking of quitting smoking  **Sample size:** 151 (intervention group-75, control group-76)  **Mean age/ age range:**  19 to 62 years  Control: 35.6 (10.3)  Intervention:36.1 (9.5) | **Modality:** text based  **Substance:**  Tobacco | **Intervention:** Dissemination of daily messages aimed at giving participants skills to help  them quit smoking.  **Control group:** A brochure that provided similar information about smoking cessation.  **Delivered by:** automated messages  **Number of sessions:** daily messages for 6 weeks  **Follow up duration:** 3 months | **Smoking cessation:** At 3 months, cessation was higher in the intervention group but no statistical difference (11% versus 5%, p=0.24)  Factors associated with cessation were sex, whereby females in intervention group were significantly more likely to have quit at 3 months than female control participants (14% vs 0%, n=0; χ  21=3.7, P=.05). and severity of smoking with light smokers (less than 20 cigarette per day) in  intervention group also were significantly more likely to have quit compared  to control participants (17% versus 0%, n=0; χ  21=5.3, P=.02) | **Recruitment:** Through in person outreach at local shopping malls, flyers, and advertisements in local newspapers. 247 were assessed for eligibility. 34% did not attend the initial enrollment meeting. Research incentives are not culturally normative; hence it was not used.  **Acceptability:**  At 4-week follow-up, 69% somewhat or strongly  liked the program and 78% were somewhat or very likely  to recommend the program | Some limitations include small sample size and technological problems such as some participants received duplicate text messages at least once  during the trial; others failed to receive some program messages. Neither receiving duplicate messages (χ  21=0.12, P=.73), or  missing 5 or more program messages (χ  21=0.75, P=.39) negatively affected quitting rates. |
| Ybarra 2013 | Turkey | **Setting:** community members  **Study population:** Adult smokers  **Sample size:** 75  **Mean age/ age range:**  37.6 years (range = 19–62 years; SD = 10.8) | **Modality:** Text messages  **Substance**  Tobacco | **Intervention:** 6 weeks of daily messages aimed at giving participants skills to help  them quit smoking.  **Control group:** None  **Delivered by:** Self-reported (75% on day 2 after quit day) The records were shared with healthcare providers after the intervention  **Number of sessions:** 6  **Follow up duration:** 12 weeks | **Abstinence:**  At 12 weeks:  13% reported  continuous abstinence since their quit date, confirmed by  carbon monoxide readings. | **Adherence/retention:**  Passive dropout was rare, there was high retention  Twelve weeks after quit day, 84% provided carbon monoxide data  **Acceptability/ satisfaction:** Among those who provided acceptability data (51%) favorable reactions to the program were observed.  69% liked the program somewhat or very much; 71% would be somewhat or very likely to recommend the program to others; 87% said the program did  not disrupt their daily schedule; 39% perceived that the program made it easier to quit smoking. No difference by gender.  89% found the messages easy to understand  15 participants asked to receive more text messages and many kept the text messages on their phones and referred back to them. | A major limitation was the absence of a control group |
| Yu 2017 | China | **Setting:** Local maternal child health centers  **Study population:**  Families with fathers who smoked at home and non-smoking mothers  of newborns  **Sample size: 342 (**114 participants to IG A, 114 participants to IG B, 114 participants to CG C)  **Mean age/ age range:**  **Husband:** 31.8 (4.5)  **Mothers:**  29.6 (3.8) | **Modality:** text message  **Substance:**  Tobacco | **Intervention 1:** (IGA) in-person counseling on the harms of secondhand smoking to infants; education on a smoke-free home and education materials including a manual with step-by-step instructions;  **Intervention 2:** (IGB) the same educational intervention and materials as intervention 1 and  received a text message intervention in the coming months, messages to the mother and her husband on the harms of SHS to the mother and the infant. The husband received additional cessation text messages to encourage him to quit smoking.  **Control group:** (CGC) Received standard care but no tobacco control and cessation counseling services  **Delivered by:** trained health care providers  **Number of sessions:** 12 text messages  **Follow up duration:** 12 months | **Abstinence**: Abstinence rates of the fathers were higher in the intervention group at 6 and 12 months (adjusted OR: 3.60, 95% CI:  1.41–9.25; p=0.008; and adjusted OR: 2.93, 95% CI: 1.24–6.94; p=0.014) respectively. | **Recruitment:**  342 households were recruited and underwent screening after randomization  **Adherence/retention:**  1GA: 103 fathers and 104 mothers present at baseline but 102 father and 104 mothers remained at 12 months  IGB: 100 fathers and 99 mothers were present at baseline but 97 fathers and 97 mothers remained at 12 months  CGC: 96 fathers and 97 mothers were present at baseline but 93 fathers and 96 mothers remained at 12 months. | Limitations include the self-reporting nature which can lead to bias, absence of biochemical verification, and non- generalization of the population |
| Zhu 2018 | China | **Setting:** Drug rehabilitation center in Shanghai  **Study population:** Male Adults with methamphetamine use disorder  **Sample size:** 40 (IG=20, CG=20)  **Mean age/ age range:**  32.70 (SD 5.27) years in the intervention group and 35.05 (SD  8.02) years in the control group. | **Modality:** Mobile application {a mobile-based program  called computerized cognition addiction therapy (CCAT)}  **Substance:**  Methamphetamine use disorder | **Intervention:** 4 weeks of CCAT with regular detoxification treatment  The app was designed to address cognitive deficits, impulse control and attentional bias.  **Control group:** Received only regular detoxification treatment and  participated in the assessments at baseline and 4 weeks later  **Delivered by:** CCAT displayed on an iPad  Initial interview and training of app by psychiatrist/ trained doctors subsequently  **Number of sessions:** 20 sessions, (5 times per week,); 60 mins per session with a 5min relaxation time afterwards for a period of 4 weeks  **Follow up duration:**  4 weeks | None reported  (Study was done in patients on inpatient detoxification) | **Recruitment:** 56 were assessed for eligibility and 40 were randomized.  **Adherence/retention:**  None was lost to follow up  **Acceptability/ satisfaction:**  Not available | Those in the intervention group had better cognitive performance after 4 weeks with changes in impulse risk-making tasks.  Some limitations were small sample size, male only population, possibility of stimulus effects |
| Bedendo 2019 | Brazil | **Setting:** Community  **Study population:**  College students in Brazil  **Sample size:** 23054 (IG- 11,529, CG-11525)  **Mean age/ age range:**18-30 years | **Modality:** Web-based  **Substance:**  Alcohol | **Intervention:**  Web-based intervention based on  personalized normative feedback.  Immediately after assessment  **Control:** assessment only  **Delivered by:** self  **Number of sessions:3**  Each lasted 5-10 minutes  **Follow up duration:**1,3, and 6 months | **Reduction in drinking:**  Those in intervention were less likely to use alcohol at months 1, 3 and 6 follow up (OR=0.71, p=0.002, OR=0.60, p<0.001 and OR=0.68, p=0.016, respectively)  This effect was moderated by motivation level whereby there was reduction in typical number of drinks in motivated students whereas low motivated student had an increase in AUDIT scores | **Recruitment:**  Done online.  **Adherence/retention:**  Less attrition in the control group compared to the intervention group (only 5.1% in control and 2.3% in intervention were present at all follow ups)  **Acceptability:** not reported | High attrition rate |
| Bedendo 2019 | Brazil | **Setting:**  Web-based among Brazilian college students  **Study population:** College students who reported alcohol use in the last three months  **Sample size:** 5,476 (PNF=  1725, NFO= 1800 CFO=1951)  **Mean age (SD)/Age range:** 18-30 | **Modality:**  Web-based  Based on personalized normative feedback (PNF)  Substance targeted:  alcohol | **Intervention:**  Full PNF:  Control:  Normative feedback only (NFO)  Consequences feedback only (CFO*)*  **Delivered by:**  Self  **Follow up duration:** follow up at 1, 3 and 6 months | **Reduction in drinking:**  Significant reduction in AUDIT scores for NFO and CFO groups compared to PNF over study period | **Recruitment:**  Was done online  45,061 participants were screened for eligibility, and 34,617 randomized  **Retention:**  Follow-up data was collected for 15.8%  Higher follow-up  among NFO (15.6%) or CFO (16.9%) compared to  PNF (15.0%) | Motivated participants more adherent to treatment (p<0.001) |
| Bernado-souza  2018 | Brazil | **Setting:** Public Brazilian Secondary Schools  **Study population:**  7th graders in three public schools  **Sample size:** 306  **Mean age/ age range:**  12.97 years (12-16 years) | **Modality:**  App on mobile phone or tablet  (Facial-aging app: “Smokerface)  **Substance:**  Tobacco | **Intervention:**  Students altered three-dimensional selfies on mobile phone or app and images projected to the class. The students then gave their perceptions.  **Control:** None  **Delivered by:** Medical students  **Number of sessions:** 1 session lasting 45 minutes  **Follow up duration:** | Not assessed. | **Treatment engagement:** All secondary school students received the intervention as outlined  **Acceptability/ satisfaction**  99.3% described the intervention as fun  88.7% reported desire to use app again  70.4% wanted the app on their phones  72.4% planned  to use the app again  98% stated they learned benefits of nonsmoking | Motivation not to smoke:  94.4% agreed/ fully agreed that the selfie motivated them not to smoke  Perceived subjective norm: positive peer pressure to remain nonsmoker was noted |
| Chen  2020 | China | **Setting:**  Community based  **Study population**  **Sample size:** 80  (IG=40, CG=40)  **Mean Age/ Age range:** 25-44 years | **Modality:**  Mobile Social network application (WeChat)  **Substance:**  Tobacco | **Intervention group:**  Received access to the full-version SCAMPI program, a Chinese-language smoking cessation program for 6 weeks  **Control group:** Had access to a static WeChat page of contacts for standard smoking cessation care**.**  Both groups received  incentive credit payments for participating  **Delivered by:**  Self    **No. of Sessions:**  Minimum once a week for 6 weeks  **Follow up:**  6 weeks | **Smoking abstinence at 6 weeks:**  Higher in intervention group: Self-reported: 38% versus 13%; Biochemically-verified 25% versus 5%)  **Number of cigarettes smoked per day**: less in intervention compared to group at 4 and 6 weeks. | **Recruitment:**  Recruitment was done online over 13-day period. 3257 were assessed for eligibility 80 participants were randomized.  **Retention**:  Self-report data at 6 weeks provided by 90% of intervention and 88% of control group.  Data on biochemically verified smoking cessation provided by 69 participants.  **Engagement:**  Average number of interactions with program higher in intervention group compared to control (82 versus 61)  **Acceptability/satisfaction:** Average rating score of 4.6 out of 5.  97% willing to introduce program to others; 64% willing to pay to use program; 97% would use program at least once in next 12 months | Some limitations include the possibility of a type 1 error as participants were not blinded |
| Marzo 2019 | Malaysia | **Setting:**  Government secondary schools  **Study population:**  Students in three government schools  **Sample size**:383  **Mean age/ age:** 13-16 years | **Modality**  Facial aging app  **Substance:**  Tobacco | **Intervention:**  Web-based mobile application called “Smokerface” based on facial aging  **Control:** None  **Delivered by:**  Self  **Sessions**: one | **Effectiveness of the app:** 87.7% and 89% agreed the app motivates them to quit smoking and not to smoke respectively. | **Satisfaction:**  85.9% found the app enjoyable and easy to handle; 83.8% would try it again in the future; 80.9% would  recommend it to others | No students had heard about the app prior to the intervention |
| Lim  2019 | Korea | **Setting:** Adults working  in an oil refining company in Korea.  **Study population**: Adults working  in an oil refining company in Korea.  **Sample size:** 60  **Mean Age:** 43.26 years (S.D = 11.19) | **Modality:**  Text messages, telephone calls and  face-to-face counseling  **Substance targeted:**  Tobacco | **Intervention group**: a smoking cessation program using weekly 10-15 minutes individual counseling (in-person for first 6 weeks and on phone for other 6 weeks)  and tailored text messaging based on SDT during 12 weeks.  **Control:**  received a smoking cessation leaflet and telephonic follow-up for 3 times.  **Delivered:** A researcher and occupational health nurse with 10-year experience  **Duration:** 12 weeks | **Smoking abstinence:**  At 6 weeks 100% in intervention and 16.1%  At 12 weeks, the abstinence rate was higher in the intervention group was 96.7% versus 13.3%,p><0.001) | **Recruitment:** 863 were assessed for eligibility, allocation was done for 60 participants  **Retention:** all participants were retained through-out 12-week study period  **Acceptability:** not reported | Autonomous regulation of intervention group increased at 6 weeks and this was maintained at 12 weeks |
| Chen 2019 | China | **Settings:** Hospital  **Study population:** Patient with alcohol dependence admitted in hospital  **Sample size:** 70 (IG=35, CG=35)  **Mean age:** 41.4 years (SD 13.3) | **Modality:**  A web-based messaging application (WeChat)  **Substance:**  Alcohol | **Intervention:** Cognitive behavior therapy via WeChat platform conducted as group therapy  **Control:** general clinical treatment with sertraline  **Delivered by:** Therapists qualified psychological counselors  **Number of sessions:**  Once a week for 12 weeks each session is 1-1.5 hours.  **Follow up:** 4. 8 and 12 weeks | **Abstinence from alcohol:**  The Severity of Alcohol Dependency Questionnaire (SADQ-C) scores in the intervention group were significantly different from those in control over the treatment period.  Rate of effectiveness higher in intervention group (83.7% versus 65.6%)  Rate of recovery was also higher in the intervention group (45.2% vs 21.9%). | **Recruitment:**  171 patients were screened Retention was 90%  Effective as there was an appreciable reduction in quantitative alcohol  **Retention:**  10% dropped out of treatment  **Acceptability:** not assessed | Self -rating anxiety scale (SAS) and self-rating depression scale (SDS) scores in both groups decreased after treatment compared to baseline  Adverse reactions were reported in the control group using pharmacotherapy (7 cases of varying symptoms) but all improved after treatment. |
| Nadasan 2017 | Romania | **Settings:** Sixteen high schools  **Study population:** Ninth graders  **Sample size:** 1369  (CG= 694 IG= 675)  **Mean age (SD):**  14.89 (0.48) for control and 14.87 (0.48) | **Treatment modality** Web based multimedia smoking prevention and cessation program  (ASPIRA)  **Substance targeted:** Tobacco | **Intervention group:** Students  received five sessions of web-based, multimedia program was designed using the social cognitive theory and the trans-theoretical model of change and it contains animations, video, and interactive activities structured in  five learning modules.  **Control:** no intervention  **Number of sessions:**  Five weekly sessions and one booster. Each session lasts for 45-50 mins  **Delivered by:**  Self on school computers  **Follow up:** | **Smoking cessation:**  Reduced smoking  initiation was observed most notably among students who were exposed to at least 75% of the ASPIRA content  Never-smoker students who attended intervention schools  were 35% less likely to report smoking initiation 6 months after  the baseline assessment | **Retention:**  More students in the intervention group were lost to follow up compared to control (30.7% vs 20.9%) | Limitations include the likelihood that the study was  underpowered to evaluate the effect of the intervention and the study had poor generalizability |
| Nandyal 2019 | India | **Setting:** Department of Psychiatry in a tertiary hospital  **Study population:**  New patients diagnosed with Alcohol dependence syndrome  **Sample size:**54  **Mean age:**40.5 years (S.D= 8.51) | **Treatment modality:** Telephone and in-person  **Substance targeted:** Alcohol | **Intervention delivery:**  Participants provided a telephone number to be contacted after 6 months  **Control:** none  **Follow up:** 6 months  **Duration/ number of sessions:** 2 assessments at baseline and 6 months | **Abstinence from alcohol:**  At 6 months: 62.7%  reported abstinence for 1 month; 27.5% were abstinent throughout the 6 months of follow up; 72% had lapses or relapses  during the follow-up period | **Recruitment:**  All the patients we approached for recruitment into the study were  willing to share their contact details and be contacted over the  phone.  **Retention:**  9.3% were lost to follow up | Limitations:  Effect of regular contact was not assessed in this study and there is the possibility of information bias |
| Onur 2016 | Turkey | **Setting:** Hospital smoking cessation unit  **Study population:**  Adult smokers who wanted to quit  **Sample size:** 436  **Mean age:**45 years (S.D= 12) | **Treatment modality:** SMS text messaging  **Substance targeted:** Tobacco | **Intervention delivery:**patients received SMS reminder s for appointment and quit date.  SMS sent on quit day and then monthly  Number of SMS increased if participant attended follow up. If not, the messages were stopped.  At 6 months patients were called.  **Control:** none  **Number of sessions:** at least four SMS per participant over 6 months  **Delivered by:**  Not reported  **Follow up:** 6 months | **Smoking abstinence:**  At 6 months:  Abstinence was 25.3%  Smoking cessation higher in those who had responded to SMS compared to those who had not responded but difference not statistically significant.  34.7% of SMS responders attended at least one follow up  no difference in follow up attendance among groups | **Engagement:**  348 were accessed by SMS while 88 were not  **Retention:** 6 months retention was 79.8% | **Limitations**  No biochemical verification was used, smoking status was given by the smokers themselves |
| Sanchez 2018 | **Brazil** | **Settings:** Nightclubs  **Study population:** Patrons of night clubs with history of drinking in the last 12 months  **Sample size:** 1057  (intervention-515, control-542)  **Mean age:**  25.8 (6.8) for the IG and 26.5 (7.4) for the CG | **Treatment modality:** Web based intervention, telephone, and face to face  **Substance targeted:** Alcohol | **Intervention delivery:**  At baseline, participants were  classified into 2 AUDIT score groups: a “high-risk” group (AUDIT 8; 44%) and a “low-risk” group (AUDIT  <8; 56%). In both groups, the intervention subgroup was exposed once to a personalized normative  feedback screen with information on the participant’s alcohol consumption and its consequences  **Duration:** Follow up at 3,6 and 12 months | **Alcohol use reduction:**  After 12 months, there were no differences between the intervention and control groups in either risk group.  In the “high-risk” group, there were significant reductions of both  the AUDIT score and the prevalence of binge drinking over time in both the control and the  intervention subgroups.  At 6 months, there was  an estimated 13% reduction in the AUDIT score in favor of the intervention group | **Recruitment:**  3063 patrons were approached and 1057 were enrolled | Other  the main limitation includes  the high rate of attrition over the 12-month period. The study  did not provide incentives to the participants, which may justify  the high rate of attrition |
| Tiburcio 2018 | Mexico | **Settings:** Two treatment centers  **Study population:** individuals who used substance seeking treatment  **Sample size:** 83 (PAADD-27, SHG +TAU =27, TAU=29)  **Mean age:** 89.2% were 18-25 years | **Treatment modality**   Web-based intervention  **Substance targeted:**  Multiple drugs such as cannabis,  cocaine, amphetamines, inhalants, hallucinogens, sedatives, and designer drugs (excluding tobacco, alcohol and opiates) | **Intervention group:**  Web-based help program for drug abuse and depression (PAADD) based on transtheoretical model of change with CBT approach  **Control 1:**  ASSIST Self-Help strategies Guide and Treatment as Usual (SHG+TAU) comprising two weeks' interaction with a counselor and 6 weeks TAU.  **Control 2:** Treatment as Usual (TAU),  Face-to face CBT  **Delivered by:** a counselor responded to the messages  **Duration:** 8weeks  **Number of sessions:** eight, each session at least one hour per week | **Abstinence:**  There was a decrease in the  average days of substance use (p < 0.001) as well as the severity of use (p < 0.00) with no significant differences among the three groups | **Recruitment:** 1558 were assessed for eligibility, 247 met the eligibility criteria, but 83 were randomized  **Retention:**  Those retained in treatment at follow-up were 33.3%, 44.4% and 34.5% of those allocated to PAADD, SHG+TAU and TAU groups respectively. | Limitations:  The difficulty of measuring participation in web-based  Treatment  Due to lack of a standard |
| Andrade 2017 | Brazil | **Setting:** Community  **Study population:**  individuals reporting alcohol use  **Sample size:**929  **Mean age:** 40 years | **Treatment modality:** Web based intervention (Berbemenos Program)  **Substance targeted:** Alcohol | **Intervention delivery:**  After completing the AUDIT, users were told which of the three risk zones they were in: low risk users (LRU; scores 0–7), hazard-  ous/harmful users (HHU; scores: 8–20) and suggestive of dependent users (SDU; scores 20 or more) and feedback given.  **Delivered by:**  Self. But a report was generated to show researchers  **Number of sessions:**  40 minutes session (online)  **Duration:** 6weeks | **Reduction in drinking:**   63.5% reported low-risk drinking levels at 6 weeks. There was a significant reduction in alcohol consumption in the HHU (62.5%) and SDU (64.5%) groups in relation to baseline | **Recruitment:**  3389 registered, 929 were eligible  users  **Retention:**  At Six weeks, those who completed the evaluation were 42 in LRU, 90 in HHU and 82 in SDU  Dropout rate was 76.9%  **Adherence:** was low since only 23.1% of those who were considered eligible (N = 929) agreed to participate and completed the first evaluation |  |
| Auguston 2017 | China | **Settings:** Community (rural and urban areas)  **Study population:** Adult population willing to quit smoking  **Sample size:**8000  (4000 in each group) | **Treatment modality:**  Text based, phone call  **Substance targeted:** Tobacco | **Intervention delivery**  High frequency text contact (HFTC) received 91 messages over 6 weeks, starting with 3 messages per day, then 2 messages per day and 1 message per day in weeks 1-2, 3-5 and 6 respectively.  **Control:**  Low frequency text contact (LFTC): 1 text message per week over the 6 weeks.  **Delivered by:** not reported  **Duration:**1, 3 and  6 months | **Smoking cessation:**  Quit rates were high in both HFTC and LFTC groups  with no significant difference between the two groups  7-day point prevalence abstinence was higher in HFTC group  compared to the LFTC group at immediately after the intervention and at each follow up with statistically significant difference. | **Feasibility:**  276000, indicated interest in participation but only the first 8000 who responded to the text message were enrolled.  Sample size achieved within one hour. |  |
| Akhu-Zaheya 2016 | Jordan | **Setting:** Outpatient clinic of a university teaching hospital  **Population:** Adult patients with cardiovascular disease who sought to quit smoking  **Sample size:** 160  **Mean age:** 54.94 (SD)= 10.86 | **Treatment modality**  Short message system (SMS)  **Substance targeted**  Tobacco | **Intervention delivery:**  received 3 messages regarding adherence to a healthy diet, medication, and smoking  cessation)  **Control:**  Control group 1: (placebo group) received general messages  Control group 2: routine care with no message  **Delivered:** not reported  **Follow up:** 3 months | **Smoking cessation:**  Those in the intervention group had more intent to quit smoking, followed by placebo group and control group, but not statistically significant (p>0.05)  There was no significant difference among groups in number of cigarettes | Feasibility and acceptability were not reported | Other outcomes were adherence to medications and dietary advice. There was a significant difference noted in the intervention group for the other outcomes -medication adherence (p=0.001) and diet (p=0.000 |
| Baldini 2018 | Brazil | **Settings:**  31 Nightclubs  **Study population:** 1057  **Sample size=** 465  IG=224  CG=241 | **Treatment modality:**  Text message  Telephone  **Substance targeted:** Alcohol | **Intervention delivery:**  Participants were given a customized normative feedback screen on the risks of alcohol use consisting of 4 parts.  Control: assessment only, no intervention  **Delivered by:** not reported  **Number of sessions:** one  **Follow up:**   6 months by email | **Reduction in alcohol use:**  Significant reduction in the practice of binge drinking in the week  estimated at 38% among participants in the intervention group after six months however, there was no significance after adjusting for age, sex and socioeconomic class. | **Recruitment:**  Of the 1832 invited to respond to online questionnaires, 86.5% accepted to participate in the study.  **Retention:**  At six-month follow-up the response rate was 50.6% and 49.4% in the intervention and control group respectively. |  |
| QUALITATIVE |  |  |  |  |  |  |  |
| Blitchstein-Winicki  2017 | Peru | **Setting:** Community  **Study population:**  Young adults interested in quitting smoking in 30days  **Sample size:** 42  Focus groups=12  Validate SMS messages=8  Pilot program=15  **Mean age/ age range:**18-25 years | Tobacco | **Modality:**  Mixed method (focus groups and RCT)  Text messages  (SMS)  Common quit practices and beliefs emerged from participants in the focus groups and interviews informed  the content, tone, and delivery schedule of the messages used in the SMS text message smoking cessation  program.  **Intervention group:** focus groups and four in-depth interviews with 12 participants  **Delivered by:** Self report provided on day 2,7 and 30. Research team members facilitating the input  **Number of sessions: 2** focus groups and 4 in depth interview Participants got a list of 60  messages between the initial meeting and a follow-up meeting 7 to 10days of engagement  **Follow up duration:** 30 days | Findings from the focus group discussions and in-depth interviews demonstrated that the majority of the  Participants who wanted to quit smoking (n=12) did not have a plan  participant was given a list of 60  messages to review between the initial meeting and a follow-up meeting 7 to 10 | **Recruitment:**  Web based advertisements were done.  639 filled the online survey form, 42 were eligible but only 35 participated  **Retention:**  was high at 93% until day 30 after quite date. |  |
| Louwagie  2019 | South Africa | **Settings:**  Three clinics in three districts in South African provinces  **Sample size:** 137    **Study population:**  **Mean age:** 39.8 years (SD 13.3) | Alcohol and  Tobacco | **Modality:**  SMS text messages and brief motivational interview  **Delivered by:** Lay hHealth workers  SMS twice weekly for 12 weeks  **Duration:** 12 weeks |  | **Satisfaction:**  All patients enjoyed the  sessions “quite a lot” or “a great deal”. Most patients  rated the intervention sessions as helpful or very helpful.  About half of the patients self-reported  reduced smoking and drinking. Most patients reported feeling better emotionally and  physically and better adherence to TB treatment. All patients liked the counselors’  style of interacting “a lot” and patients ascribed many positive attributes to the | several patients did not like the counseling sessions being recorded Lay  health workers also felt recorders hindered free participation |
| Valente 2018 | Brazil | **Setting:** Parents of adolescents  **Study population:** Parents of adolescents, aged 10 to 18 years, who sought a drug prevention program and did not report a drug use problem  Sample size: 26 (intervention=13, control=13)  **Mean age/ age range: Mean age** 43.6 years | Any psychoactive substance drug used | **Modality:** Telephone call  **Intervention group:** (BMI group) received Brief motivational interviewing  **Control group:** PE group received psychoeducation  **Delivered by:** Consultants monitored by doctoral and masters’ students from the healthcare system  **Number of sessions:** one  **Follow up duration:** day1,7,14,28 | Parents in the intervention group improved their parenting styles compared with the control group  The difference between the groups were not significant  However, BMI has a greater potential for reducing the risk factors and increases the protective factors of drug use involved in the educational attitudes of the parents | **Recruitment:**  Publicized through the internet, television, newspaper and letters to schools.  29 parents were assessed for eligibility  **Adherence/retention:**  26 parents enrolled in the study and 15 parents completed the follow-up assessment at 28days 7 losses in the follow up of the PE and 4 losses in the BMI group  **Feasibility:**  Assessed based on the number of families enrolled in the study and the number who completed the prevention intervention | Short duration of follow up and the small sample size were some limitation  Partnering with institutions and building on cross- institutional support |
| Do 2020 | Vietnam | **Setting:** community health centers  **Study population:** male smokers with or without intention to quit  **Sample size:71 (**37 intention to quit, 34 no intention to quit)  **Mean age (SD):** 42.2 | **Modality:**  Text message    **Substance targeted**  Tobacco | 10 focus groups  5 had smokers with intention to quit in 30 days  5 had smokers with no intention to quit  Each session lasted 90-120 minutes  Semi structured interview guide with questions on: interest in text message intervention, suggestion on content of message; preference for structure and timing of message; ideas to make intervention user friendly; other outcome related to mindfulness treatment  Included a video with introduction to mindfulness (behavior theory that the intervention was to be based on) | Level of interest in text message:  Overall high interest in those with intention to quit. Those with no intention to quit perceived message as not helpful to quit  Concerns about the program:  Majority said the program should be different from other educational campaigns for it to be effective  Expressed concerns that there may be too many message  Perception that text message would not be suitable for older people  Those with basic phones were less interested in reading messages  Suggestion for message content:    Information on negative effects and harm to self and others  Quitting tips and encouragement\strategies to cope with craving  Information on available treatment such as medications  including images and videos  Frequency of message delivery:  Those with intention to quit wanted more message  Average 1-2 messages per day  Frequency to be reduced with time:  higher at the beginning  Timing of message delivery:  Most preferred early morning and evening time  (participants reported these times as period most likely to smoke**)**  Interactive messages  perceived as helpful by majority  Suggestion to include keywords in intervention | Perception about mindfulness-  Acceptable as a tool to help relax or reduce stress  Perception about mindfulness smoking cessation-  Almost all participants thought it would be helpful |  |
| Other study designs: Secondary analysis e.t.c |  |  |  |  |  |  |  |
| **Author, year** | **Country** | **Sample characteristic** | **Treatment modality**  **Substance targeted** | **Data collection** | **Feasibility /acceptability outcomes** | **Other outcomes** |  |
| Han 2018 | China | **Setting:** Three MMT clinic  **Study population:** adults using drugs in past 30 days  **Sample size:**  **7**5 (50 intervention, 25 control)  **Mean age (SD):** 41.6 (8.0) | **Modality:**  Mobile App  (S-Health)  **Substance targeted**  Multiple  Heroin, cocaine, methamphetamine, marijuana, MDMA | Ecological momentary assessment (EMA)- using the app  Urine drug screen (UDS)  Life experience timeline assessment (LET) | Correspondence between the 3 methods of assessment  There was poor agreement throughout study period  Between EMA and LET- 67%,79%,72%,86%  Between EMA and UDS-51%, 65%, 62%, 72% from week 1 to 4  Daily survey data received:  Average 49.3%  Reduction overtime  From 1^st^ week to 4^th^ week  59.1%, 48.9%, 48%,41.1% respectively | This study was a secondary analysis of data by Liang et al 2018, with the aim of examining the feasibility of the intervention |  |
| Borzekowiski 2016 | India | **Setting:** school, college, and popular neighborhoods  **Study population:** Adults in the community  **Sample size:**205  **Duration:** 10 days | Mobile app-ecological momentary assessment (EMA to examine real-time tobacco use and exposure to social and environmental cues    **Substance:** Tobacco | Staggered time periods,  participants were randomly signaled 5–8 times per day for ten consecutive days during waking hours (defined as  8 am to 10 pm) on their mobile phone, which instructed  them to take the momentary prompt (MP) survey | **Abstinence:**  33.7-37.6% used tobacco at least once during study period. |  |  |
| Cartujano-Barrera 2020 | Mexico | **Study setting:**  Mexican smokers  **Study population:**  Adults interested in quitting smoking  **Sample size:** 40  **Mean age (**SD): 38.1 (10.7)  **Duration:**  12 weeks | **Modality:** a secondary data analysis  of a single-arm feasibility study in Mexico.    An mHealth intervention enhancing nicotine replacement therapy usage and adherence  **Substance:**  Tobacco  **Delivered by**: self-reported daily patch usage  Tobacco treatment specialist | This was a secondary data analysis  of a single-arm feasibility study  Participants’  interactions with the program via text messages  were monitored throughout the entire 12-week  intervention and retrospectively analyzed.  At 12-weeks follow-up, the  following were assessed: cessation outcomes and  pharmacotherapy use, delivery, and side effects. | During  the 12-week study period, participants sent 620 messages to the program (mean=20.6, SD=18.34) of which 12.7% were  related to nicotine NRT. Three themes were identified in the messages related to NRT: enthusiasm, instructions, and side effects.  At 12 weeks, 40%  of participants reported using NRT <75% of the days.  biochemically verified abstinence in 30%  at 12 weeks |  |  |
| Nadasan 2019 | Romania | **Settings:** School based in sixteen high schools  **Study population:** Ninth graders  **Sample size:** 1369  (CG= 694 IG= 675)  **Mean age (SD):**  14.89 (0.48) for control and 14.87 (0.48) | **Modality:** web-based program  **Substance:**  Tobacco | Secondary analysis of data for participants in the intervention group of a RCT who completed follow up (65%) | **Feasibility:**  Average number of education activities was completed was 79.5%  68.3% had high exposure (more than 75% of intervention content) with only 20.1% having exposure below 50%  Level of exposure to the program was significantly associated with, father’s low level of education, participant brother not smoking and never smoked and considering the health of others as a reason not to smoke |  |  |
